# Substoichiometric Hsp104 regulates the genesis and persistence of self-replicable amyloid seeds of Sup35 prion domain

**DOI:** 10.1016/j.jbc.2022.102143

**Published:** 2022-06-14

**Authors:** Sayanta Mahapatra, Anusha Sarbahi, Priyanka Madhu, Hema M. Swasthi, Abhishek Sharma, Priyanka Singh, Samrat Mukhopadhyay

**Affiliations:** 1 Centre for Protein Science, Design and Engineering, Indian Institute of Science Education and Research (IISER) Mohali, Punjab, India; 2 Department of Biological Sciences, Indian Institute of Science Education and Research (IISER) Mohali, Punjab, India; 3 Department of Chemical Sciences, Indian Institute of Science Education and Research (IISER) Mohali, Punjab, India; 4 CSIR-Institute of Microbial Technology (IMTECH), Chandigarh, India

**Keywords:** amyloids, protein aggregation, chaperones, intrinsically disordered proteins, yeast prion, AFM, atomic force microscopy, GdmCl, guanidinium hydrochloride, HRP, horseradish peroxide, Ni-NTA, nickel-nitriloacetic acid, PBST, PBS with Tween-20, PEP, phosphoenolpyruvate, PK, pyruvate kinase, PQC, protein quality control, TEV, Tobacco Etch virus, ThT, thioflavin-T, Trp, tryptophan

## Abstract

Prion-like self-perpetuating conformational conversion of proteins is involved in both transmissible neurodegenerative diseases in mammals and non-Mendelian inheritance in yeast. The transmissibility of amyloid-like aggregates is dependent on the stoichiometry of chaperones such as heat shock proteins (Hsps), including disaggregases. To provide the mechanistic underpinnings of the formation and persistence of prefibrillar amyloid seeds, we investigated the role of substoichiometric Hsp104 on the *in vitro* amyloid aggregation of the prion domain (NM-domain) of *Saccharomyces cerevisiae* Sup35. At low substoichiometric concentrations, we show Hsp104 exhibits a dual role: it considerably accelerates the formation of prefibrillar species by shortening the lag phase but also prolongs their persistence by introducing unusual kinetic halts and delaying their conversion into mature amyloid fibers. Additionally, Hsp104-modulated amyloid species displayed a better seeding capability compared to NM-only amyloids. Using biochemical and biophysical tools coupled with site-specific dynamic readouts, we characterized the distinct structural and dynamical signatures of these amyloids. We reveal that Hsp104-remodeled amyloidogenic species are compositionally diverse in prefibrillar aggregates and are packed in a more ordered fashion compared to NM-only amyloids. Finally, we show these Hsp104-remodeled, conformationally distinct NM aggregates display an enhanced autocatalytic self-templating ability that might be crucial for phenotypic outcomes. Taken together, our results demonstrate that substoichiometric Hsp104 promotes compositional diversity and conformational modulations during amyloid formation, yielding effective prefibrillar seeds that are capable of driving prion-like Sup35 propagation. Our findings underscore the key functional and pathological roles of substoichiometric chaperones in prion-like propagation.

Protein misfolding results in the deposition of proteinaceous β-rich amyloid aggregates and is associated with a range of fatal neurodegenerative diseases (1, 2). Prions belong to one of the subclasses of amyloids that can exhibit a self-perpetuating conformational conversion. They can migrate from a small infected patch to the distal parts of the neuronal tissues resulting in adverse cellular consequences leading to neurodegeneration (3). The prion-like mechanism has also been proposed for other amyloidogenic proteins such as α-synuclein, tau, amyloid β (Aβ), Huntingtin, p53, and so forth (4, 5, 6, 7, 8). The prion-like spreading in the brain is thought to involve the preformed self-replicable amyloid entities called the propagons or seeds that are considered as the minimum units of amyloid infections. Aging increases the frequency of these events as the protein quality control (PQC) system faces challenges (9). The PQC system comprising a sophisticated network of proteins, called the chaperones, is not only devoted to the proper folding of nascent polypeptide chains but also guides the unfolded and misfolded proteins to attain the native three-dimensional shape by inhibiting their aberrant aggregation and eliminating irreversibly aggregated proteins (10, 11, 12). Disaggregases (Hsp110 in higher eukaryotes; ClpB in *Escherichia coli*; Hsp104 in yeast) belong to an important class of chaperones that are involved in the ATP-dependent and cochaperone-regulated disassembly of aggregated proteins that bypass the other surveillance of the PQC system (13, 14, 15, 16, 17). In aged neurons, however, one of the critical manifestations of the PQC dysfunction is the lower expression of these disaggregases such as Hsp110, Hsp70, and so forth (18). The insufficiency of the disaggregases has been linked with the amyloid-promoting propensity and its fatal consequence (19).

A functional prion protein (Sup35) that is beneficial to yeast serves as an excellent model to develop the prion concept as well as to elucidate the role of disaggregases in the prion-like transmission of several disease-associated amyloids. This is due to the following reason (20). Firstly, due to its super-structural resemblance with disease-linked amyloids that exhibit a prion-like propagation. Secondly, the distinct prion phenotypes [*PSI*^*+*^ and *psi*^-^] and the prion strains can be recapitulated by the protein-only transmission using *in vitro* generated amyloids. Interestingly, these traits exhibit a dose-dependence with respect to the cellular disaggregase machinery of yeasts namely, Hsp104, a hexameric AAA+ ATPase that controls the crossgenerational non-Mendelian inheritance of [*PSI*^*+*^] phenotype and is reminiscent of the neuron-to-neuron transmission of self-replicable amyloid seeds (21, 22, 23). A concerted activity of cochaperones such as Hsp70 or Hsp40 with Hsp104 is shown to be important for the amyloid remodeling activity *in vivo* and *in vitro*. However, Hsp104 alone can autonomously disaggregate Sup35 amyloids *in vitro* (13, 24). Additionally, *in vivo* studies showed that the overexpression of Hsp104 alone was sufficient to remodel Sup35 aggregates (25, 26). Whereas, in the case of other yeast prions such as Ure2p, the role of Hsp70 and Hsp40 is thought to important for prion propagation (27). Therefore, at least for Sup35 amyloids, Hsp104 is believed to be the principal chaperone that governs prion propagation (13, 24). At higher concentrations, Hsp104 dissolves the aggregates up to the noninfectious level and impairs the passage of the prion phenotype resulting in curing of the [*PSI*^*+*^] phenotype (28). Also, the genetic or chemical inactivation of Hsp104 hinders the propagation of [*PSI*^*+*^] phenotype due to the unavailability of enough prefibrillar seeds that are generated from matured fibrils by Hsp104 (29, 30). Though generation and persistence of prefibrillar amyloids as the seeds for the successful prion-like infection is critical as matured amyloid fibrils show limited infective potential due to their fewer ends of polymerization and lower cytoplasmic diffusibility (31, 32, 33, 34). Understanding the underlying mechanism of prion formation and propagation at low concentrations of Hsp104 is important for the studies related to the disaggregase underexpression during aging and its link with the elevated risk of prion-like amyloid colonization. The studies have suggested that the substoichiometric Hsp104 accelerates the fibrillation that minimizes the existence of prefibrillar aggregates resulting in less efficient fibrillar seeds (35). Also, the chance of generating prefibrillar seeds indirectly through disaggregating fibrils by Hsp104 in such low concentrations is very nominal (24). Therefore, collectively these observations do not fully explain the critical aspect of the abundance of prefibrillar amyloid species in the presence of low concentrations of Hsp104 that is crucial in the self-templating cascade of prions.

In this work, we aim at deciphering the molecular mechanism behind the feasible generation of prefibrillar amyloids as effective seeds by only Hsp104 at low concentrations, in the absence of Hsp70 or Hsp40, through *in vitro* recapitulation of the cellular scenario of yeasts in a minimalistic approach. We used the NM domain of the *Saccharomyces cerevisiae* translation termination factor, Sup35. The NM domain is intrinsically disordered in the (monomeric) nonprion form and comprises the N-terminal part abundant in polar uncharged amino acids (glutamine, asparagine, and tyrosine) and a highly charged middle region (M) (Fig. 1*A*). The NM domain of Sup35 is necessary and sufficient to recapitulate all the characteristics of the prion state, and therefore, represents a prion determinant in yeast. Using substoichiometric ratios of Hsp104, we detected a pronounced kinetic alteration of the NM aggregation behavior that supported not only the rapid generation of seeding-competent prefibrillar amyloids but also ensured the prolonged persistence of these species before their recruitment into matured amyloid fibers. Additionally, we were also able to capture conformationally distinct, Hsp104-remodeled NM species that exhibit a much higher seeding potential.

**Figure 1:**
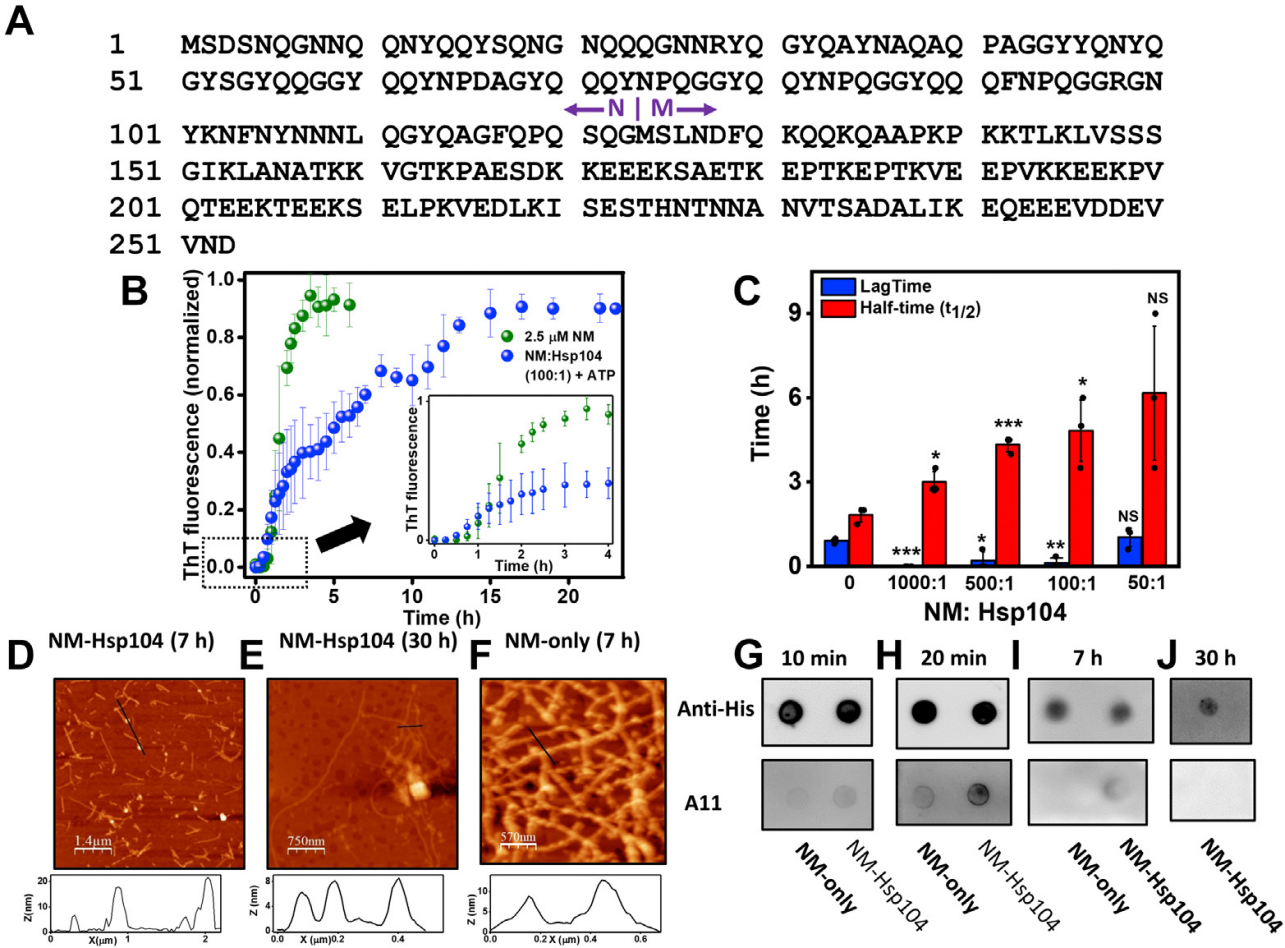
**Hsp104 alters the fibrillation kinetics of NM.***A*, the amino acid sequence of Sup35NM showing the putative boundary between the N- and M-domain. *B*, normalized thioflavin-T (ThT) fluorescence kinetics of NM (2.5 μM) without or with Hsp104 (0.025 μM) and ATP (5 mM) during amyloid formation (stirred at 80 rpm at room temperature). The kinetics of the first 5 h from the commencement of the reactions are shown in the inset. The individual ThT kinetics of NM-Hsp104 aggregation is shown in Fig. S2, *A*–*C*. SDs were estimated from three independent replicates (n = 3). Additional three independent replicates were also provided in Fig. S2, *D*–*F*. *C*, the lag time and t_1/2_ of the NM aggregations without or with various substoichiometric ratios of Hsp104. The lag times were retrieved by fitting the first 6 h fluorescence intensities to sigmoidal function, and t_1/2_ were determined from the time points when the normalized fluorescence intensities reached 0.5. The SDs were calculated from three independent experiments (n = 3). ∗∗∗*p* < 0.001, ∗*p* < 0.05, ∗∗*p* < 0.01, NS (not significant) for NM:Hsp104 1000:1, 500:1, 100:1, 50:1, respectively, compared to the lag time of aggregation reactions without Hsp104. ∗*p* < 0.05, ∗∗∗*p* < 0.001, ∗*p* < 0.05, NS for NM:Hsp104 1000:1, 500:1, 100:1, 50:1, respectively, compared to the half-time of the aggregation reactions without Hsp104 (One-way ANOVA). *D* and *E*, AFM images of NM amyloids (2.5 μM monomers) showing the oligomers and protofibrils with the height of ∼20 nm and 7 nm, respectively, in the presence of Hsp104 (0.025 μM), plus ATP after 7 h (*D*) and fibrils with the height ∼7 nm after 30 h (*E*) and also after 2 h and 25 h (Fig. S3, *D* and *E*) from the commencement of the reactions. *F*, AFM image of NM fibrils (2.5 μM monomers) formed after 7 h of aggregation in the absence of Hsp104 with the *height* ∼9 nm. *G*–*J*, samples from the NM aggregation reactions without or with Hsp104 (NM:Hsp104 100:1) and ATP were spotted on nitrocellulose membrane after (*G*) 10 min, (*H*) 20 min, (*I*) 7 h, and after (*J*) 30 h for NM-Hsp104 aggregation only, from the commencement of the reactions and dot-blotted with the anti-His and A11 antibodies. AFM, atomic force microscopy; Trp, tryptophan.

## Results

### Hsp104 modulates the NM assembly kinetics

We first carried out the amyloid formation kinetics at a low micromolar protein concentration in the absence of Hsp104 using a well-known amyloid reporter, namely, thioflavin-T (ThT). The aggregation of NM (2.5 μM) proceeded *via* typical nucleation-dependent polymerization kinetics possessing a lag phase of approximately 50 min, an assembly phase, and a saturation phase (Fig. 1*B*) (36, 37). In order to investigate the effect of the low concentrations of Hsp104 in NM assembly, we performed the aggregation kinetics in the presence of Hsp104 at several substoichiometric ratios containing ATP and an ATP regeneration system after checking the activity of Hsp104 using luciferase reactivation assay (Fig. S1). We observed rapid oligomerization of NM and shortening of the lag phase in the presence of Hsp104, an observation that is consistent with the previous study. At the lowest concentration of Hsp104 (NM:Hsp104 = 1000:1), the lag phase is almost abolished (Figs. 1, *B* and *C* and S2*H*). Interestingly, the shortening of the lag phase in the presence of a low concentration of Hsp104 is associated with a delay in the assembly phase. This observation indicated that the assembly and maturation of Hsp104-induced early species get retarded in a dose-dependent manner (Figs. 1*C* and S2*I*). As a control experiment, we performed NM aggregation just in the presence of ATP and ATP regeneration system and observed no significant change in the NM aggregation profile in the absence of Hsp104 (Fig. S2*G*). Next, in order to directly visualize the nanoscale morphology, we carried out atomic force microscopy (AFM) imaging, which revealed the appearance of spherical oligomers in both the aggregation reactions after 25 min (Fig. S3, *B* and *C*). In the Hsp104-mediated NM aggregation reaction, a mixture of spherical oligomers and protofibrils were observed that matured into longer fibrils after 30 h from the commencement of the reaction. In contrast, in the absence of Hsp104, we observed primarily matured fibrils at a much earlier time point (7 h) (Fig. 1, *D*–*F*). The length of matured fibrils formed in the absence or presence of Hsp104 was in the micrometer range, whereas protofibrils were much shorter in the submicrometer range (Figs. S3, *F*, *G* and S6*B*). We validated the early oligomerization by Hsp104 by probing both the aggregation reactions at the early time points by an oligomer-specific antibody such as the A11 antibody (38, 39, 40). More intense signals from the spots corresponding to the Hsp104-mediated aggregation reaction confirmed an increased oligomerization in the early time points compared to the NM-only aggregation reaction (Fig. 1, *G* and *H*). Next, we wanted to test if the observed spherical aggregates formed after 7 h during the NM-Hsp104 reaction retained the characteristics of amyloid oligomers (Fig. 1*D*). We were able to detect the A11-positive signal, albeit weaker, indicating the existence of a smaller fraction of the oligomeric species in the presence of Hsp104 but not in its absence. This weak A11-reactivity in the presence of sub-stoichiometric Hsp104 disappeared after 30 h, presumably due to the complete conversion of the oligomeric species into matured fibrils (Fig. 1,*I* and *J*). Therefore, early spherical oligomers and/or short (submicron) protofibrils can possibly represent crucial prefibrillar species. Together, this set of results showed that Hsp104, at a substoichiometric concentration, accelerates the early oligomerization events but decelerates the growth kinetics allowing a prolonged persistence of prefibrillar species (oligomers and protofibrils) before they mature into amyloid fibers.

### The role of Hsp104-mediated disaggregation in the NM assembly kinetics

**Figure 2:**
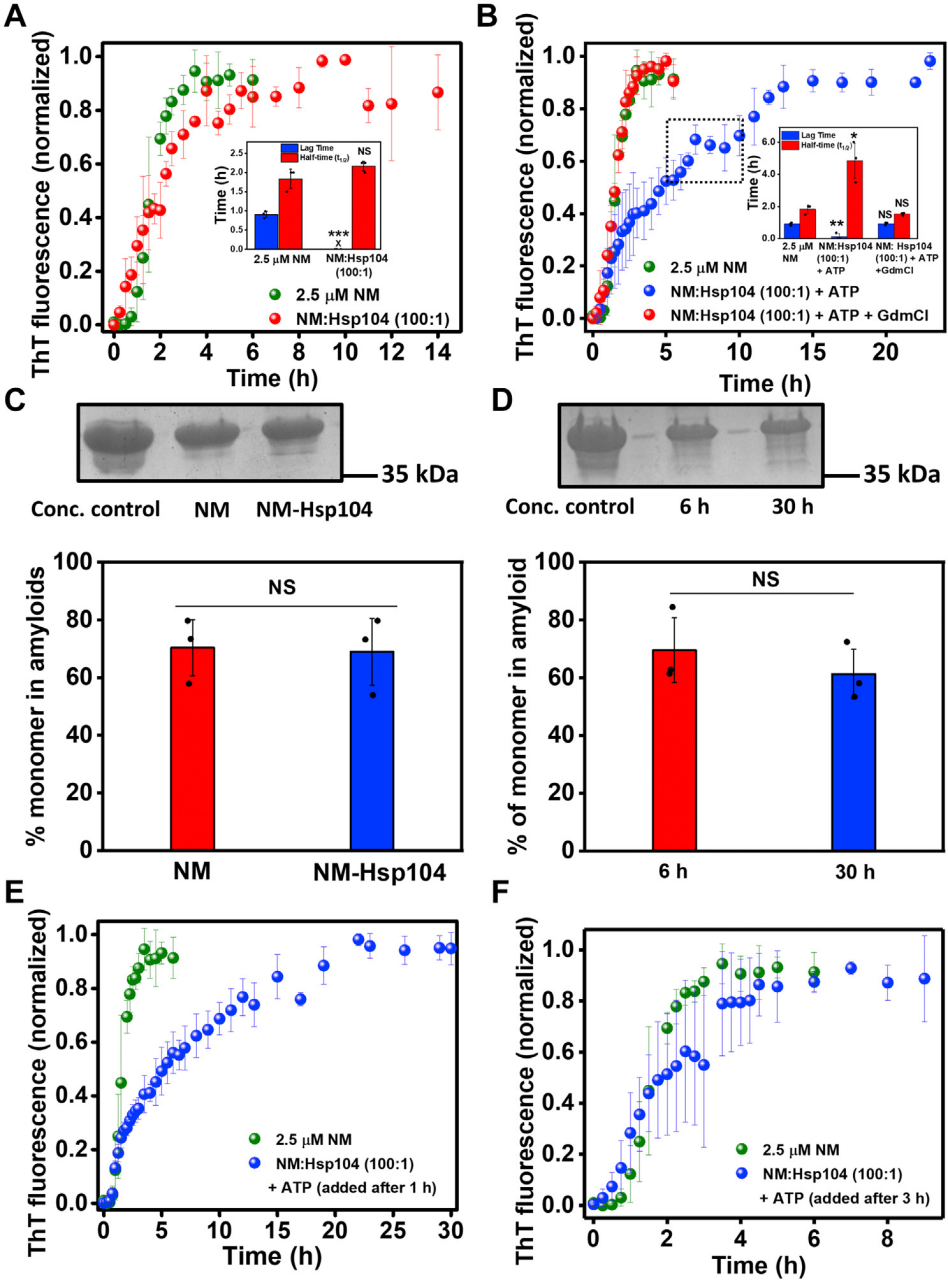
**The disaggregase activity of Hsp104.***A*, normalized ThT fluorescence kinetics of rotated (80 rpm) NM (2.5 μM) aggregation without or with Hsp104 (0.025 μM). The lag time and t_1/2_ are retrieved from three independent experimental replicates (n = 3), ∗∗∗*p* < 0.001, NS for lag time and t_1/2_, respectively, compared to the NM-only aggregation. (One-way ANOVA) (inset). *B*, normalized ThT fluorescence kinetics of rotated (80 rpm) NM (2.5 μM) aggregation without or with Hsp104 (0.025 μM), plus ATP and with Hsp104 (0.025 μM) and ATP, in the presence of GdmCl (3 mM) in the assembly buffer that alone does not alter the NM aggregation (Fig. S4*A*). Representative ‘halt’ in the NM-Hsp104 aggregation is marked. The lag time and t_1/2_ are retrieved from three independent experimental replicates (n = 3). NS, NS for lag time and t_1/2_ of NM-Hsp104-GdmCl aggregation and ∗∗*p* < 0.01, ∗*p* < 0.05 for lag time and t_1/2_, respectively, for NM-Hsp104 aggregation compared to NM-only aggregation (One-way ANOVA) (inset). *C*, the relative quantification by ImageJ software of NM monomers retrieved from the amyloids formed from the rotated (80 rpm) polymerization of NM (2.5 μM) without or with Hsp104 (0.025 μM) and ATP for 6 h or 30 h, respectively, with respect to the concentration control in SDS-PAGE. SDs were estimated from three independent replicates (n = 3), NS compared to NM samples (One-way ANOVA). The uncropped gel is show in Fig. S8*A*. *D*, the relative quantification by ImageJ software of NM monomers retrieved from the amyloids in the aliquots of rotated (80 rpm) polymerization of NM (2.5 μM) with Hsp104 (0.025 μM) and ATP, aliquoted before the ‘halt’ marked in Figure 2*B* (6 h from the commencement of the aggregation) and at the end of the polymerization (30 h from the commencement of the aggregation). SDs were estimated from three independent replicates (n = 3), NS compared to NM samples (One-way ANOVA). The uncropped gel is shown in Fig. S8*B*. *E* and *F*, normalized ThT fluorescence kinetics of rotated (80 rpm) NM (2.5 μM) aggregation without or with Hsp104 (0.025 μM), plus ATP, introduced after (*E*) 1 h (f) 3 h from the commencement of the reaction. SDs were calculated from three independent experiments (n = 3). GdmCl, guanidinium hydrochloride; ThT, thioflavin-T.

Next, we asked whether the modulation in the aggregation kinetics by Hsp104 is due to its specific disaggregase activity or a passive perturbation in the NM polymerization by this chaperone. In order to distinguish between these two possibilities, the aggregation reaction of NM monomers with Hsp104 was set up in the absence of ATP, as Hsp104 is ATP-dependent amyloid disassembling machinery, and we did not observe any measurable change in the aggregation kinetics (Fig. 2*A*). We also performed NM aggregation with Hsp104 and ATP but in the presence of a millimolar concentration of guanidinium hydrochloride (GdmCl) that acts as a potent inhibitor of Hsp104 by preventing its ATP hydrolysis-dependent disaggregase activity (41, 42, 43). In this case, we did not observe any changes in the aggregation profile, suggesting a coordinated role of ATPase and disaggregase activities of Hsp104 in altering the NM aggregation behavior (Fig. 2*B*). On this basis, we further tested if there was a different extent of monomer recruitment in amyloids in NM-only and Hsp104-mediated NM assembly due to their pronounced kinetic dissimilarities. However, when we retrieved the high molecular weight aggregates in the pellet fraction after the completion of the aggregation reactions by high-speed centrifugation and monomerized them using the denaturant, we observed similar intensities of the NM band on SDS-PAGE for NM-only and Hsp104-mediated NM aggregations. This indicates the recruitment of nearly the same fraction of NM monomers into aggregates in both types of reactions (Fig. 2*C*). We were also able to copellet amyloid-bound Hsp104 with the pelleted amyloids that indicated the amyloid-Hsp binding (Fig. S3*H*). By the scrutiny of the aggregation profiles, we noticed some temporary halts resulting in separable biphasic kinetics (marked in Fig. 2*B*) in the amyloid formation in NM-Hsp104 aggregation reactions, more pronounced in the relatively higher ratios of Hsp104, and often these halts in the aggregation are reported to be associated with the fresh recruitment of monomers on preformed amyloid surfaces *via* the secondary nucleation mechanism (Fig. S2*I*) (44). To assess the possibility of secondary nucleation here, we aliquoted the NM-Hsp104 reaction mixture just before the halt and after the completion of the aggregation and then the retrieved amyloids were monomerized using the denaturant. The nearly identical monomeric NM band intensities in the SDS-PAGE in both prehalt and posthalt samples ensured no additional recruitment of monomers (Fig. 2*D*). Also, such Hsp104 dose-dependent halts were absent both in the absence of ATP that drives the Hsp104 disaggregase machinery and in the presence of 3 mM concentration of GdmCl that acts as a small-molecule inhibitor of Hsp104 (Fig. 2, *A* and *B*). Together, this set of data suggested the specific ATP-dependent, GdmCl-sensitive, enzymatic activity of Hsp104 in the modulation of the NM assembly kinetics resulted from an intricate balance between the intrinsic propensity of the amyloidogenic intermediates to mature into higher-order amyloid species and Hsp104-mediated amyloid disaggregation. We, however, would like to point out that we cannot completely rule out the possibility of secondary nucleation that might partially contribute to the observed multiphasic kinetics.

### The role of Hsp104 in amyloid maturation

During the budding process of yeasts, the daughter cells receive a fraction of the cytoplasm from their mothers containing the preformed amyloid species of various molecular weights. Inefficient conversion of these low molecular weight aggregates into matured fibers is critical to maintain and propagate the amyloid-linked [*PSI+*] phenotype because of the limited infectivity of the mature fibers. Therefore, in order to delineate the putative role of Hsp104 in the persistence of low molecular weight amyloid species, we introduced a low concentration of Hsp104 with ATP at the late lag phase (0.5 h after commencement of the reaction) and at the early log phase (1 h after commencement of the reaction) of NM aggregation reactions (Figs. 2*E*, S4, *E* and *F*). The kinetics revealed that Hsp104 delayed the maturation of these already formed particles to the higher-order amyloids resulting in the persistence of shorter protofibrils that eventually converted into matured fibrils at a much later time as observed by AFM (Fig. S4, *B*–*D*). In contrast, when Hsp104 and ATP were introduced at the end of the elongation phase (3 h after commencement of the reaction) of the NM aggregation, no significant modulation in the aggregation kinetics was observed. This observation revealed the inability of the low concentration of Hsp104 to manipulate the higher molecular weight aggregates (Fig. 2*F*). Together, these results tell us to surmise that the low concentration of Hsp104 enhanced the abundance of low molecular weight species as opposed to mature fibrils not only by amending the *de novo* aggregation but also decelerating the conversion of the preformed amyloid species to higher molecular weight fibers.

### The seeding capability of the Hsp104 remodeled amyloid

**Figure 3:**
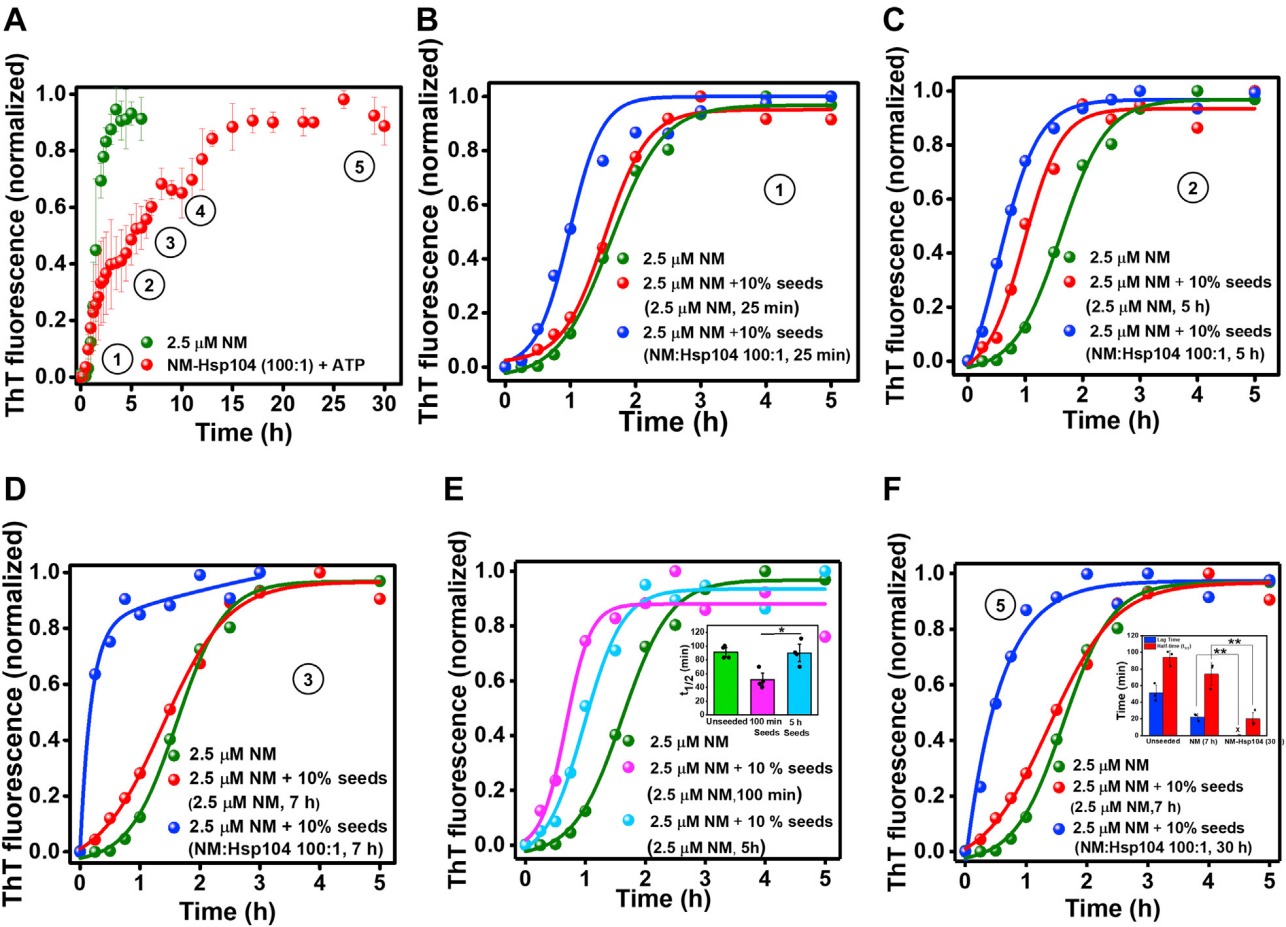
**Seeding by NM and NM-Hsp104 amyloids.***A*, normalized ThT fluorescence kinetics of rotated (80 rpm) NM (2.5 μM) aggregation without or with Hsp104 (0.025 μM), plus ATP, and aliquots were withdrawn from these reactions as seeds at the indicated time points (*circled numbers*) and introduced to the fresh aggregation of NM (2.5 μM) in assembly buffer containing GdmCl (3 mM). *B*–*D*, representative normalized ThT fluorescence kinetics of rotated (80 rpm) NM (2.5 μM) aggregation without or with 10% (w/w) seeds of NM-Hsp104 or NM aggregation which were aliquoted after (*B*) 25 min, (*C*) 5 h, (*D*) 7 h, and after 10 h (Fig. S5*A*) from the commencement of the aggregation reactions. *E*, representative normalized ThT fluorescence kinetics of rotated (80 rpm) NM (2.5 μM) without or with 10% (w/w) seeds from NM aggregation that were aliquoted after 100 min and 5 h showing the t_1/2_ of the unseeded and seeded aggregations. SDs were calculated from four individual experiments (n = 4), ∗*p* < 0.05 with respect to 100 min seeds and 5 h seeds (One-way ANOVA) (inset). *F*, representative normalized ThT fluorescence kinetics of rotated (80 rpm) NM (2.5 μM) aggregation without or with 10% (w/w) seeds from NM or NM-Hsp104 aggregation that were aliquoted after 7 h or 30 h, respectively. Lag time and t_½_ of the unseeded and seeded aggregation reactions are shown in the inset. SDs were calculated from three individual experiments (n = 3), ∗∗*p* < 0.01 and ∗∗*p* < 0.01 for NM-Hsp104 seeds compared to NM seeds for lag time and t_1/2_, respectively (One-way ANOVA) (inset). GdmCl, guanidinium hydrochloride; ThT, thioflavin-T.

In order to continue the cycle of typical prion-like amplification for the inheritance of the [*PSI+*] phenotype, both the abundance and the effective seeding ability of prefibrillar particles are vital. To shed light on this critical aspect of prion inheritance, we studied the seeding capability of the amyloid species of Hsp104-mediated NM aggregation by aliquoting preformed amyloids from the reaction mixture at different time points (Fig. 3*A*). The morphological and immunoblot characteristics are summarized in Table 1. The NM-Hsp104 aggregation mixture was introduced into the fresh NM-only polymerization reactions in the buffer containing GdmCl to suppress the effect of Hsp104. This allowed us to study the effect of Hsp104-remodeled aggregates in seeding the NM-only aggregation kinetics without the influence of Hsp104 in seeded reactions. This set of studies showed that the amyloid prefibrillar entities of Hsp104-induced NM aggregation had a greater potential to accelerate the fresh NM fibrillization compared to the amyloids of NM-only aggregation reactions as reflected in the lag time and the half-time (t_½_) of the seeded kinetics (Figs. 3, *B*–*D* and S5, *A*–*D*). Moreover, the seeding ability of the particles of NM-Hsp104 aggregation reactions that aliquoted after 25 min demonstrated the early appearance of the seeding-competent amyloid species in Hsp104-mediated NM aggregation compared to the NM-only aggregation reaction. This observation indicated that the seeding ability was associated with the composition of the amyloid particles of the NM-Hsp104 aggregation, which was enriched in the precursor of fibrillar amyloids of NM having a better seeding potential as opposed to matured fibrils. We also surmise that the intermediate NM particles demonstrated higher seeding potential than matured fibers (Fig. 3*E*). However, intriguingly, even the NM-Hsp104 fibrils also displayed better seeding potential compared to the NM fibrils (Fig. 3*F*). Taken together, the greater capability of Hsp104-designed NM fibrils to catalyze the NM assembly than the typical NM fibrils established the fact that the reason behind their better seeding ability was not only related to the polymerization hierarchies and sizes of the amyloids present in the seed aliquots but also the conformational attribute of Hsp104-remodeled fibrils. Therefore, we postulated that Hsp104, at substoichiometric concentrations, can craft structurally altered amyloids that can autocatalytically accelerate a fresh aggregation reaction more efficiently owing to their distinct amyloid packing. Next, we aimed at distinguishing the conformational characteristics of NM-only and NM-Hsp104 amyloids by following an array of distinct biochemical and biophysical readouts by using matured NM-only or NM-Hsp104 fibrils from the saturation phase of aggregation reactions that were devoid of any detectable oligomers.

**Table 1:** A summary of morphological and immunoblot characteristics of the seeds

Seeds aliquoted after	Seeds from NM aggregation	Seeds from NM-Hsp104 aggregation
25 min	Less A11-positive oligomers	More A11-positive oligomers compare to the NM-only aggregation
7 h	Micrometer long fibrils and no A11-positive oligomers	Submicrometer long protofibrils with a small fraction of A11-positive oligomers
30 h	NA	Micrometer long fibrils and no A11-positive oligomers

### Hsp104 induces amyloid structural diversity

**Figure 4:**
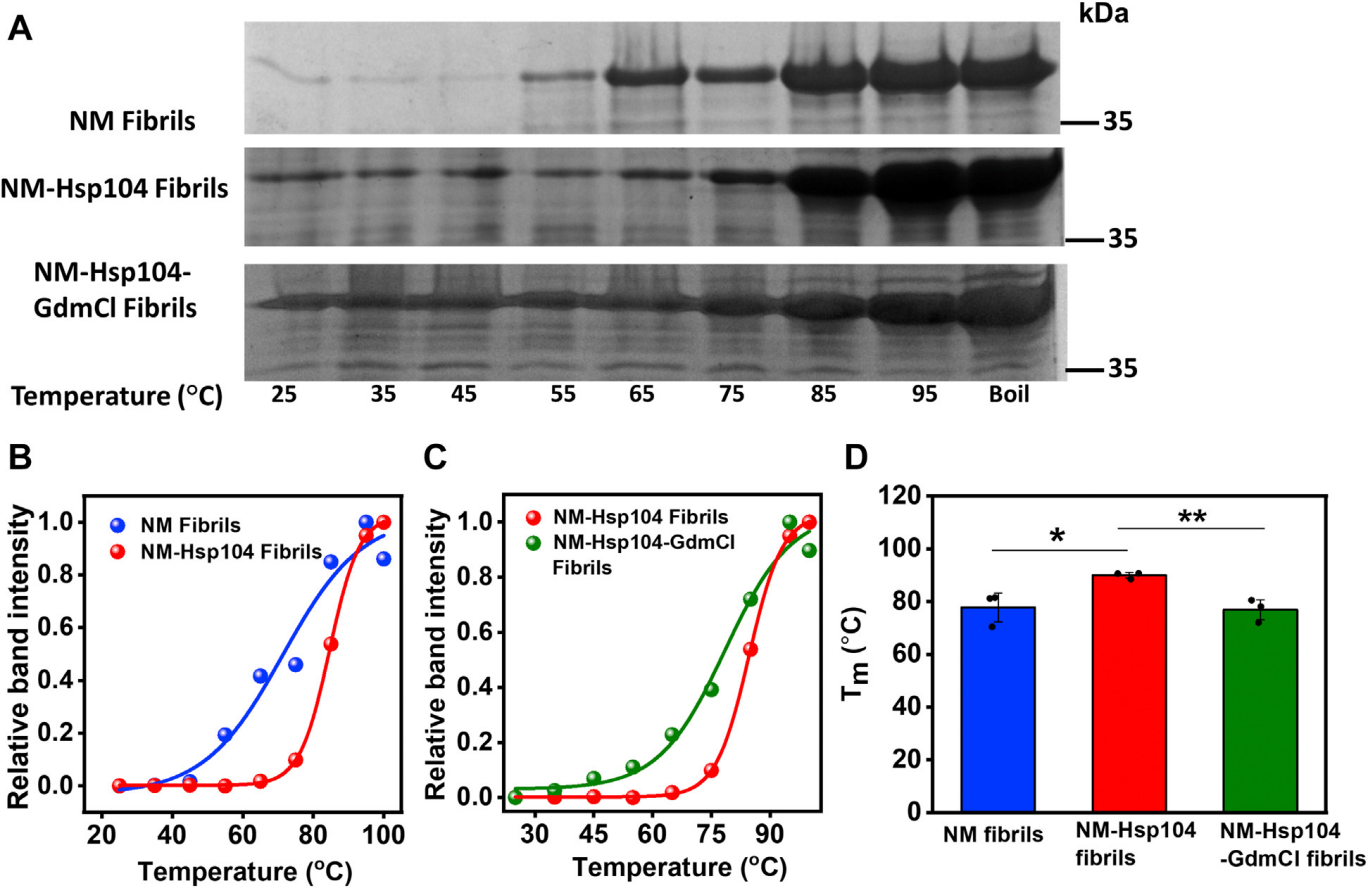
**Thermal stability of amyloids.***A*, SDS-PAGE gel image of concentrated fibrils formed from the NM monomers (2.5 μM) that fibrillized in the absence or presence of Hsp104 (0.025 μM) and ATP and also in the presence of Hsp104 (0.025 μM), ATP, and GdmCl (3 mM), which were then heated with SDS-PAGE loading dye (2% SDS) for 5 min at indicated temperatures. *B* and *C* the fitted band intensity of the monomers melted from (*B*) NM and NM-Hsp104 fibrils, (*C*) NM-Hsp104, and NM-Hsp104-GdmCl fibrils with temperature to sigmoidal functions after relatively quantified using ImageJ software. Uncropped gels are shown in Fig. S8, *C*–*E*. *D*, melting temperature (*T*_m_) of NM, NM-Hsp104, and NM-Hsp104-GdmCl fibrils retrieved from the midpoint of the sigmoidal melting curve. SDs were calculated from three individual experimental replicates (n = 3), ∗*p* < 0.05 (NM and NM-Hsp104 fibrils), ∗∗*p* < 0.01 (NM-Hsp104 and NM-Hsp104-GdmCl fibrils) (One-way ANOVA). GdmCl, guanidinium hydrochloride.

In order to monitor the amyloid structural diversity, we first studied the SDS solubility of NM-only and NM-Hsp104 aggregates. The SDS-induced thermal denaturation was earlier used to identify the structural diversity by monitoring the dissimilar thermal stability of two different yeast prion strains generated *in vitro* (45). We fibrillized NM without or with Hsp104 at the substoichiometric ratio and then treated these fibrils with 2% SDS and heated from 25 °C to 100 °C. Then, we quantified the monomeric fraction derived from this treatment on SDS-PAGE (Fig. 4). The temperature dependence of the monomeric fraction exhibited a sigmoidal profile showing an increase in the monomeric population with increasing temperature in the two types of fibrils. The dissimilar melting temperatures (*T*_m_) of NM-only and NM-Hsp104 fibrils revealed altered thermal stability arising due to their distinct supramolecular structural differences despite having similar nanoscale morphologies. However, NM-Hsp104 fibrils prepared in the presence of 3 mM GdmCl that acts as a small-molecule inhibitor of Hsp104 by binding with the nucleotide-binding domains exhibited thermal stability that is similar to NM-only fibrils. These results together indicated that the disaggregation-competent Hsp104 induces an altered amyloid packing of NM compared to pure NM fibrils.

### Hsp104 alters fibril fragility and protease digestion profiles

**Figure 5:**
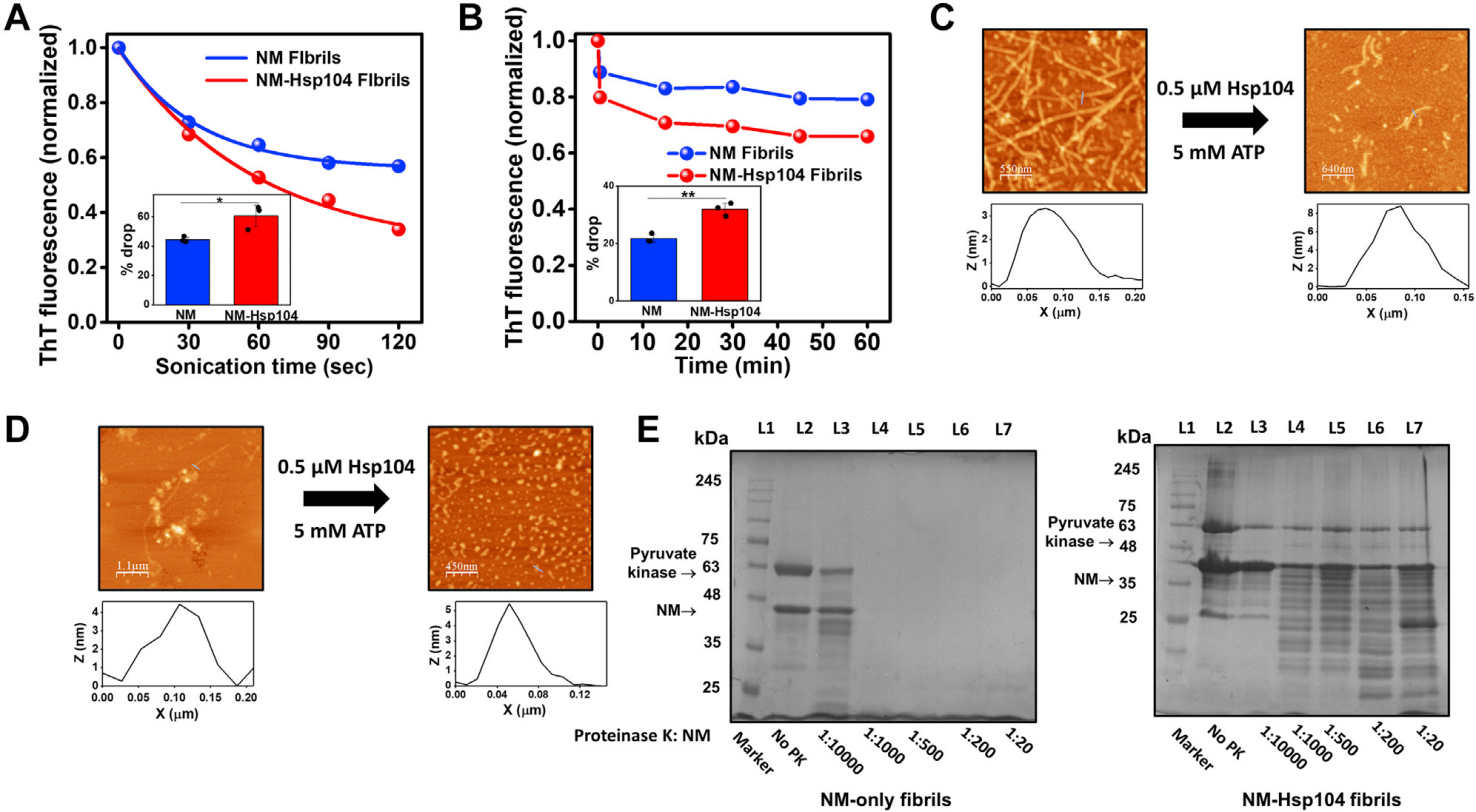
**Hsp104 alters the fragility and protease digestion profile of amyloids.***A* and *B*, monomeric NM (2.5 μM) was fibrillized without or with Hsp104 (0.025 μM), plus ATP, and then disaggregated by two methods. *A* representative disaggregation kinetics using the ultrasonic sound pulse of amplitude 5 for several pulses of 30 s and the drop in the ThT fluorescence was recorded after each pulse. The ThT fluorescence intensities were normalized with respect to the initial ThT fluorescence intensity. The extent of disaggregation at 80 rpm was estimated from three experimental replicates to calculate the SD (n = 3), ∗*p* < 0.05 compared to NM fibrils (One-way ANOVA) (inset). The AFM images of sonicated fibrils and their *length* estimations are provided in Fig. S6, *A*–*D*. *B*, representative disaggregation kinetics by Hsp104 (0.5 μM), ATP (5 mM), and drop in ThT fluorescence with time at 80 rpm were recorded. The ThT fluorescence intensities were normalized and the extent of disaggregation was estimated from three independent replicates (n = 3), ∗∗*p* < 0.01 compared to NM fibrils (One-way ANOVA) (inset). *C*, AFM images of NM-only fibrils before and after 15 min of disaggregation by Hsp104 (0.5 μM) and ATP (5 mM) showing the *height* profiles. *D*, AFM images of NM-Hsp104 fibrils before and after 15 min of disaggregation by Hsp104 (0.5 μM) and ATP (5 mM) showing the *height* profiles. *E*, the concentrated fibrils formed from monomeric NM (2.5 μM) without or with Hsp104 (0.025 μM), plus ATP were incubated at 37 °C for 30 min with multiple concentrations of proteinase K followed by the SDS-PAGE analysis and stained with Coomassie dye. Bands corresponding to the NM monomers are marked with *black triangles*. Pyruvate kinase was added after the fibrillation in the case of NM-only aggregation to make the reaction mixtures comparable to the NM-Hsp104 aggregation reaction. Uncropped gels are shown in Fig. S8, *F* and *G*. AFM, atomic force microscopy; ThT, thioflavin-T.

To further support our assertion that Hsp104 induces conformationally altered fibrillar architecture, we intended to distinguish NM-only and Hsp104-mediated fibrils by their fragility. We fragmented the NM-only and Hsp104-designed NM fibrils using three different ways: fragmenting fibrils by ultrasonic sound, by incubating fibrils at a high concentration of Hsp104, and by keeping the fibrils under unagitated conditions at room temperature (RT) for 24 h. Irrespective of the fragmentation method, the ThT fluorescence exhibited dissimilar kinetics for NM-only and NM-Hsp104 fibrils, indicating their altered structural packing in these two types of fibrils because the distinct supramolecular arrangements within the fibrils can give rise to the observed kinetic difference in the fragmentation propensity (Figs. 5, *A*–*D* and S6*E*). Next, in order to further probe into the kinetic stability of these two types of NM fibrils, we performed protease digestion assays.

Despite having a generic cross β-sheet secondary structure in different amyloid variants of a given protein, the varied supramolecular packing and nanoscale organization of the monomeric polypeptide units in the polymeric architecture lead to altered sensitivity to proteolytic digestion (46).We incubated NM-only and NM-Hsp104 fibrils with an increasing concentration of proteinase K and observed a different digestion pattern on SDS-PAGE and by the Western blot analysis. The binding of Hsp104 to the fibrils may only minimally control the digestion of NM-Hsp104 fibrils as we used a substoichiometric concentration of Hsp104 for these experiments. We would like to mention here that even in the absence of proteinase K for both NM-only and NM-Hsp104 samples, we observed a few lower molecular weight bands on the gel possibly due to an esterase-like activity of amyloids (47). In the case of NM-only fibrils, an intact monomeric NM band was visible only in the presence of the lowest amount of protease, as the higher ratios of proteinase K completely digested NM into low molecular weight peptides. In contrast, the NM fibrils designed by Hsp104 showed much more resistance toward protease digestion, and the appearance of the undigested monomeric NM bands and partially digested NM peptides at relatively higher concentrations of proteinase K suggested the existence of a more resistant amyloid core in NM-Hsp104 fibrils compared to NM-only fibrils (Figs. 5*E* and S6*F*).

### Site-specific conformational mobility distinguishes the types of amyloids

**Figure 6:**
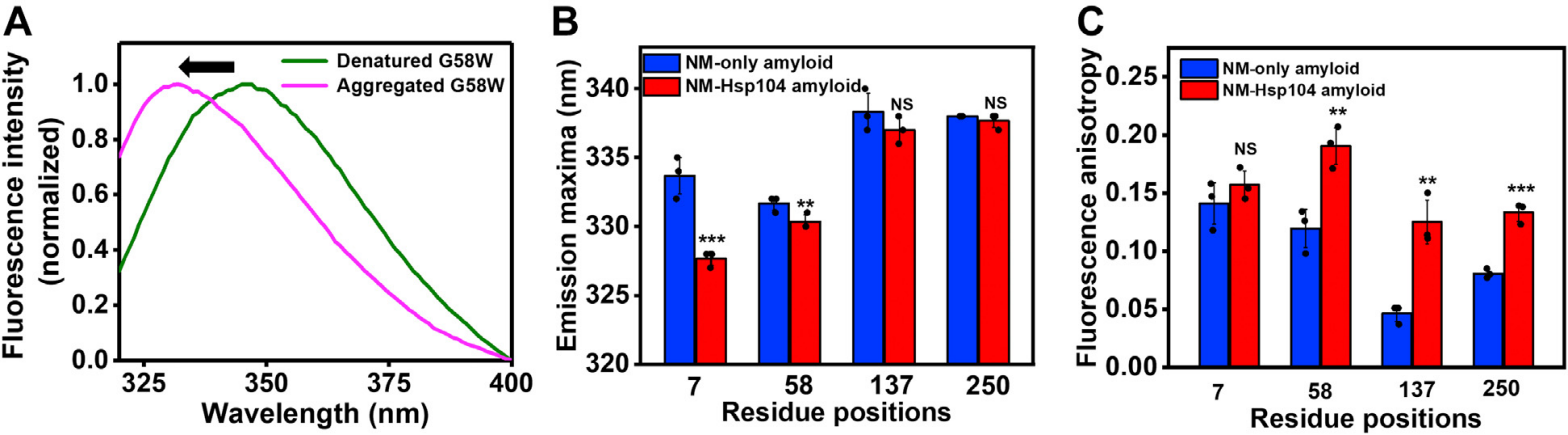
**Site-specific conformational mobility.***A*, normalized Trp fluorescence spectra of residue position 58 depicting the blueshift (*black arrow*) upon conversion into the amyloids. *B*, Trp emission maxima of different residue positions in two different amyloid forms, NM, and NM-Hsp104 (NM: Hsp104 100:1). The excitation and emission slit widths were 1.75 and 6 nm, respectively. SDs were estimated from three different experimental replicates (n = 3), ∗∗∗*p* < 0.001, ∗∗*p* < 0.01, NS, NS (One-way ANOVA) for locations 7, 58, 137, 250, respectively, with respect to the NM-only amyloids. The individual emission spectra are represented in Fig. S7, *A*–*C*. *C*, steady-state fluorescence anisotropies of different residue positions in two amyloid states (NM and NM-Hsp104, NM:Hsp104 100:1). SDs were estimated from three independent replicates (n = 3), NS, ∗∗*p* < 0.01, ∗∗*p* < 0.01, ∗∗∗*p* < 0.001 (One-way ANOVA) for locations 7, 58, 137, 250, respectively, with respect to the NM-only amyloids. Trp, tryptophan.

Next, in order to directly capture the site-specific structural information within the amyloid conformers, we performed site-specific fluorescence polarization anisotropy measurements that allowed us to monitor the conformational dynamics in two types of NM fibrils. NM and Hsp104 sequences are devoid of any tryptophan (Trp) residue, and therefore, we used single-Trp variants spanning the NM polypeptide sequence (Fig. 1*A*). We then compared the Trp emission spectra in the monomeric and amyloid states and observed a blueshift for all residue positions in the aggregated form with respect to the denatured monomeric form, which is in line with our earlier studies (Fig. 6*A*) (48). In both types of fibrils, NM-only and NM-Hsp104, the extent of the blueshift was more for N-domain residues than for M-domain residues, indicating a solvent-excluded environment in the N-domain. Moreover, on the comparison between the two different fibrils, we observed a greater extent of blueshift for NM-Hsp104 fibrils. This blueshift is more pronounced in the N-terminal segment containing residue 7 (Fig. 6*B*). This finding indicated that the NM sequence, especially the N-terminal part, experiences more solvent protection in NM-Hsp104 fibrils compared to NM-only fibrils. Next, we performed the steady-state fluorescence anisotropy measurements that report the site-specific rotational flexibility of Trp in the NM sequence (49, 50). Upon conversion to the amyloids, N-domain residues exhibited higher anisotropies indicating the more restricted rotation due to the preferential recruitment of the N-domain into the amyloid core. In contrast, the M-segment (residue 137) exhibited a much lower fluorescence anisotropy in both types of amyloids (Fig. 6*C*), an observation that is consistent with previous structural studies on NM amyloid, indicating the higher flexibility of the M-segment that is not sequestered into the amyloid core. The significantly high anisotropy for NM-Hsp104 fibrils at this residue location possibly indicated the Hsp104-binding induced restricted rotation that got partially relieved when a lower dose of Hsp104 was used during fibrillation. These NM-Hsp104 fibrils showed an intermediate anisotropy value with respect to the values of NM-only fibrils and NM-Hsp104 fibrils where NM: Hsp104 was 100:1 (Fig. S7*H*). Additionally, higher fluorescence anisotropy in all residue locations for NM-Hsp104 fibrils compared to NM-only fibrils indicated more closely packed ordered organization in NM-Hsp104 fibrils corroborating our protease digestion results.

Next, in order to further test the hypothesis of Hsp104-mediated remodeling of amyloid conformers for preformed low molecular weight NM species, we introduced substoichiometric Hsp104 at the early log phase of the NM fibrillation reaction, that is, 1 h after the commencement. Our site-specific fluorescence studies on such amyloids exhibited dynamical characteristics that are similar to NM-Hsp104 amyloids in which Hsp104 was introduced at the beginning of the amyloid reaction (Fig. S7, *D* and *E*). These NM-Hsp104 particles formed by introducing Hsp104 at the early log phase also displayed an enhanced seeding capability compared to NM-only fibrils (Fig. S7, *F* and *G*). These results suggested that the Hsp104-induced remodeling is similar when it is added either in the beginning or at the early log phase of the aggregation reaction. Taken together, a series of biochemical and biophysical studies supported our hypothesis that apart from the composition of the seeds enriched in prefibrillar aggregates, NM-Hsp104 aggregates, in contrast to NM-only aggregates, comprise an altered and more ordered amyloid packing that allows them to display their enhanced autocatalytic self-templating ability.

## Discussion

Irrespective of the precise mechanistic differences between the prion-like propagation of neurotoxic species by the transmission of seeds across the cellular membrane and the crossgenerational cytoplasmic inheritance of amyloid-associated phenotypic traits by fungal prion particles in the continuous stream of cytoplasm from mother to daughter yeast cells, the successful expedition of infectious amyloid particles into the uninfected cells revolves around the autocatalytic behavior of prefibrillar amyloids (31, 32, 33, 51). In this study, by *in vitro* reconstruction, we were able to recapitulate two crucial proprion aspects of substoichiometric Hsp104. Firstly, low concentrations of the Hsp104 facilitated the production of seeding-competent amyloid entities and decelerated the conversion of these prefibrillar species into the matured fibrils. Secondly, in addition to the kinetic modulations, we were able to identify conformational remodeling in the amyloids by the Hsp104 contributing to the better seeding potential of these species. Additionally, contrary to the view of irreversible hindrance in the fibrillation of various intermediate amyloid species of different amyloidogenic proteins including full-length *S. cerevisiae* Sup35 by Hsp104, here we observed a tradeoff between the two opposing factors namely, the intrinsic nature of NM amyloids to polymerize into higher molecular weight aggregates and the ATP-dependent GdmCl-sensitive disaggregation by Hsp104 that delayed but not inhibited the fibrillation process (52, 53). Furthermore, the critical balance between these two mutually opposing factors resulted in the observed halts resulting in apparent biphasic kinetics as GdmCl, the Hsp104-inhibitor, eliminated the halts. In contrast, the higher concentrations of Hsp104 increased the duration of kinetic halts. These halts allowed a prolonged persistence of the prefibrillar particles, pivotal for the propagation of the prion phenotype. We would like to note here that the early part of the aggregation kinetics is in line with the previous study using untagged NM (Fig. S3*A*) (35). In our current study, we were able to resolve the unique and previously undetected kinetic signatures due to the *in situ* kinetic design that permitted us to acquire a larger number of data points over a much longer observation time and with a higher time resolution.

**Figure 7:**
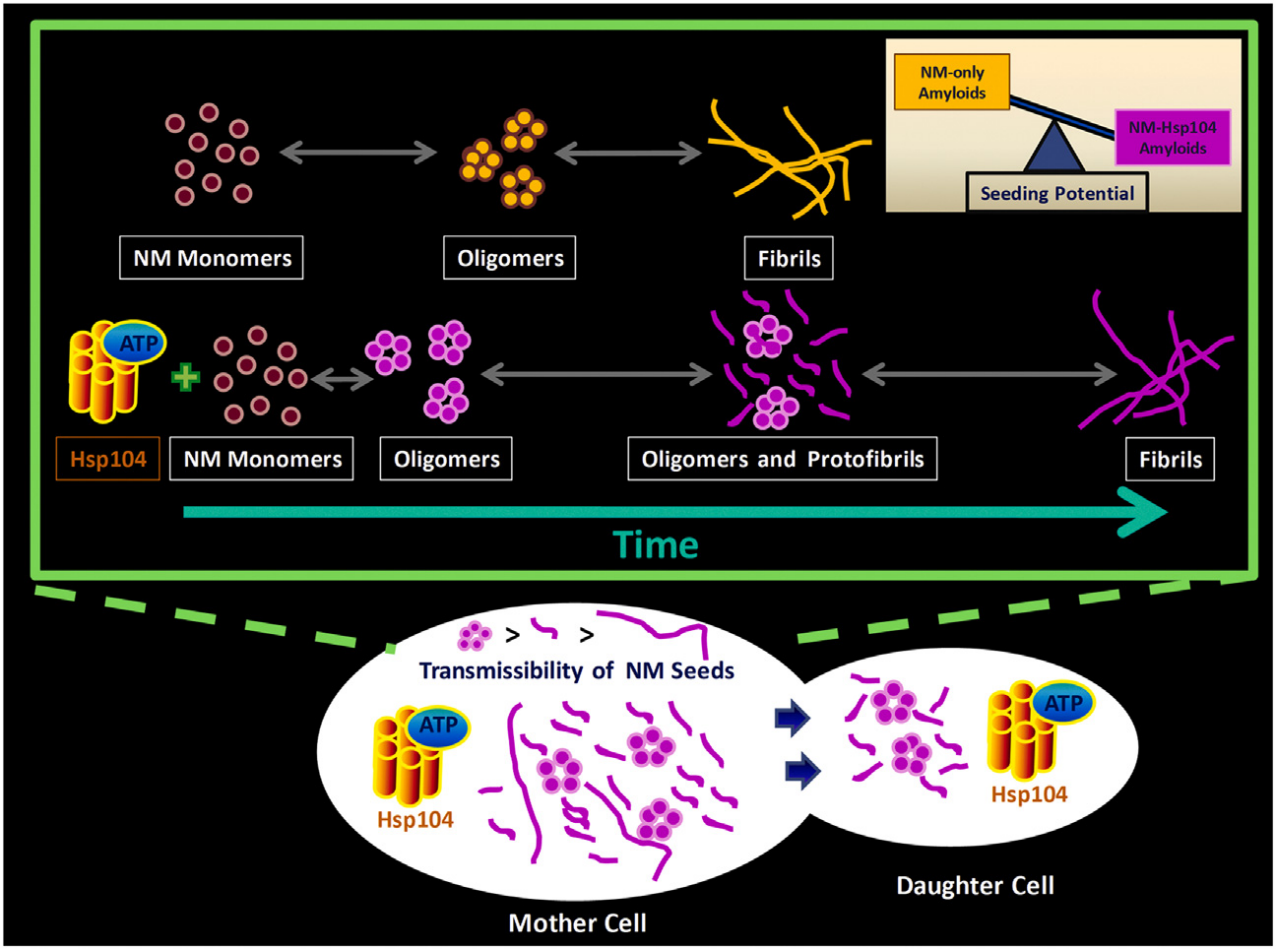
**Theproposedmodel for the NM aggregation in the presence of substoichiometric Hsp104.** Hsp104 with ATP ensures the abundance of highly transmissible (31) prefibrillar amyloids species (oligomers and protofibrils but not long and matured fibrils) that shows the greater seeding potential than amyloid fibrils generated in the absence of Hsp104.

Apart from ensuring the abundance of highly transmissible, seeding-proficient, fibrillar precursors instead of having fewer and longer matured fibrils with limited transmissibility, Hsp104, at low substoichiometric concentrations, creates seed units possessing a better seeding potential due to their unique conformational characteristics. The remodeling of the NM monomers or early soluble species, by substoichiometric Hsp104, aided the early oligomerization of NM, leading to the bypass of the lag phase. This remodeled NM species probably also gave rise to the altered building blocks for conformationally unique NM-Hsp104 fibrils. Our model in Figure 7 depicts these molecular events demonstrating the rapid formation and slow maturation of lower-order aggregates into the higher-order aggregates in the presence of Hsp104, but in the absence of its cochaperone Hsp70 and Hsp40, possibly leading to the abundance and preferential cytoplasmic transmission of conformationally distinct, seeding-proficient oligomers and protofibrils as opposed to matured fibrils (31). In this study, we probed this conformational identity using a host of biochemical tools that revealed Hsp104-mediated NM fibrils are more stable and contain a more ordered amyloid core compared to NM-only aggregates.

In accordance with our biochemical findings, location-specific spectral shifts and dynamics revealed *via* fluorescence anisotropy measurements indicated more buried locations and higher polypeptide ordering in Hsp104-mediated fibrils. The amyloidogenic N-segment is known to constitute the amyloid core, whereas, the charged M-region possesses some conformational flexibility (48, 54, 55) as also detected by our fluorescence anisotropy measurements. Hsp104 is known to interact with the M-region of NM, and therefore, can result in the binding-induced restriction in the conformational dynamics of the M-region (43, 56). However, higher anisotropy in the N-domain in the presence of Hsp104 is likely to be caused by the higher structural ordering of the amyloid core in Hsp104-mediated NM fibrils. We would like to note that in contrast to previously reported yeast prion strains, in our case, more stable and ordered NM-Hsp104 fibrils were found to be more fragile to fragmentation by Hsp104, which indicates an intriguing interplay of stability and fragility that can have a diverse phenotypic outcome (45, 57, 58, 59, 60).

In summary, our findings indicate the pro-[*PSI*^*+*^] nature of the substoichiometric concentrations of Hsp104, which aid the generation and persistence of the highly transmissible prefibrillar seeding-proficient amyloid species that do not readily transform into matured fibers. The Hsp104-mediated hindrance in the conversion of low molecular weight aggregates into matured fibers is crucial in maintaining and propagating the amyloid-linked [*PSI*^+^] phenotype in yeast. Although our study aimed at delineating the effect of Hsp104 alone in the propagation of prion phenotype, we would like to state that the association of other cochaperones may further regulate the disaggregation machinery resulting in a coordinated modulation in prion generation and propagation in yeast. Our results have broader implications in both functional and pathological prion-like mechanisms in higher organisms. The disaggregase activity and kinetic modulation of Hsps on a wide range of proteins leading to the generation and persistence of conformationally distinct self-replicating amyloid species can potentially underlie a consensus mechanism for the generation and colonization of the toxic, transmissible, neuropathological prefibrillar amyloids. The cascade of molecular events further tuned by the cochaperones and cellular microenvironments can promote the prion-like propagation strategies for the distal invasion in the aged brains having impairment in protein homeostasis characterized by the scarcity of disaggregases.

## Experimental procedures

### Materials

Hepes, magnesium chloride hexahydrate, sodium phosphate dibasic dihydrate, tris (hydroxymethyl) aminomethane (Tris), β-mercaptoethanol, ATP disodium salt hydrate, DTT, and ThT were bought from Sigma. GdmCl, proteinase K, and urea were procured from Amresco. Ammonium sulfate, imidazole, lysozyme, SDS, EDTA, and potassium chloride was bought from HIMEDIA. Potassium hydroxide, sodium chloride, sodium hydroxide, glycerol, A-11 antiamyloid oligomer antibody, methanol, horseradish peroxide (HRP)–conjugated goat anti-rabbit antibody was procured from Merck. IPTG and antibiotics (chloramphenicol and ampicillin) were purchased from Gold Biocom. Enhanced chemiluminescence kit, HRP-conjugated rabbit antimouse antibody, was obtained from Thermo Fisher Scientific. Nickel-nitriloacetic acid (Ni-NTA) column and Q-Sepharose were from GE Healthcare Lifesciences. Phosphoenolpyruvate (PEP) and pyruvate kinase (PK) were procured from Roche Diagnostics.

### Methods

#### Expression and purification of Sup35NM

C-terminal hexa-histidine recombinant Sup35NM proteins were overexpressed in BL21 (DE3)/pLysS cells using IPTG and then from the harvested cells proteins were extracted; the extracted proteins were subjected to first Ni-NTA purification in the gradient of imidazole and further from a Q-sepharose column using the gradient of sodium chloride. The detailed protocol is described by us (48).

#### Expression and purification of Hsp104

A modification of a previous protocol was used (61). N-terminal His_6_-tag recombinant Hsp104 pPROEX-HTb-Hsp104 of *S. cerevisiae* were overexpressed in BL21(DE3) RIL *E. coli* cells using 1 mM IPTG as inducer at 15 °C for 14 h. Harvested cells suspended in chilled 10 ml lysis buffer (40 mM Hepes–KOH pH 7.4, 500 mM KCl, 20 mM MgCl_2_, 2.5% (w/v) glycerol, 20 mM imidazole) were incubated in 4 °C with lysozyme (2 mg/ml) followed by sonication. The cell debris was removed by centrifugation at 11,500 rpm for 30 min, and the supernatant was subject to Ni-NTA purification using the gradient of imidazole. After Ni-NTA purification, the eluant was buffer exchanged with the (20 mM Tris–HCl pH 8, 0.5 mM EDTA, 5 mM MgCl_2_, 50 mM NaCl) using MWCO 30,000 Amicon Ultra (Millipore) 15 ml centrifugal concentrator units. The protein further purified using the Q-sepharose column using the gradient of NaCl, and the eluant was further buffer exchanged with the cleavage buffer (20 mM Hepes–KOH pH 7.4, 140 mM KCl, and 10 mM MgCl_2_) using the concentrator unit mentioned previously. Recombinant Tobacco Etch virus (TEV) protease carrying a hexa-histidine tag was used at a ratio His_6_-Hsp104: TEV protease (15:1) to cleave the histidine (His_6_) tag of the Hsp104 at 30 °C for 1 h. The cleaved His_6_ tags of Hsp104, the uncleaved His_6_-Hsp104, and the histidine-tagged TEV protease were removed by binding them with the Ni-NTA resin, and in the flow-through, the pure Hsp104 with no histidine tags were collected and stored in a storage buffer (20 mM Hepes–KOH pH 7.4, 140 mM KCl, and 10 mM MgCl_2_, 1 mM DTT, 0.5 mM EDTA) at −80 °C until further use. The luciferase reactivation assay confirmed the activity of Hsp104. Luciferase (80 nM) in Tris–HCl buffer, pH 7.4, was denatured at 45 °C for 7 min, chemiluminescence were recorded before and after denaturation. The chemiluminescence was also recorded for denatured luciferase without or with bichaperone (Ssa1 1 μM, Ydj1 1 μM) or trichaperone preparations (Ssa1 1 μM, Ydj1 1 μM, Hsp104 6 μM) in the presence of the Luciferase assay reagent, 1 mM ATP, 1 mM DTT, and ATP-regeneration system in Tris–HCl buffer, pH 7.4. The extent of recovery of luciferase was calculated by the percentage of chemiluminescence generated with respect to the native luciferase, which was more in the case of trichaperone machinery compared to the bichaperone machinery. This validated the activity of Hsp104 used in this study.

### Amyloid aggregation reactions

For the setting up of aggregation reactions, methanol precipitated NM was dissolved in 8 M urea (20 mM Tris–HCl buffer, pH 7.4) for 3 h at RT. Monomerized protein was first passed through a 100 kDa filter to remove any preexisting aggregates if present, and subsequently, the filtrate was concentrated using a 3 kDa filter before the aggregation reaction. The concentrated monomers of NM were further centrifuged at 13,000 rpm for 15 min at RT, after which the supernatant was added such that its final concentration is 2.5 μM in assembly buffer (40 mM Hepes–KOH pH 7.4, 150 mM KCl, 20 mM MgCl_2_, 1 mM DTT, 10 μM ThT) without or with Hsp104 and 5 mM ATP and ATP-regeneration system (20 mM PEP and PK (15 μg/ml) at RT under stirring at 80 rpm using the magnetic beads. ThT fluorescence was monitored RT by exciting at 450 nm, and the fluorescence emission was recorded at 480 nm. Hsp104 influenced NM aggregation reactions were also carried out under the same aggregation conditions and assembly buffer independently with ATP, ATP-regeneration system, and 3 mM GdmCl in the assembly buffer and also in the absence of ATP and ATP-regeneration system.

### Seeded aggregation reactions

Seeds of NM were generated by incubation of monomerized NM (2.5 μM) protein in assembly buffer (40 mM Hepes–KOH pH 7.4, 150 mM KCl, 20 mM MgCl_2_, 1 mM DTT) without or with Hsp104 (0.025 μM), ATP (5 mM), and ATP-regeneration system (20 mM PEP and 15 μg/ml PK) at RT under stirring at 80 rpm using magnetic beads. The resulting amyloid seeds from NM or Hsp104 controlled NM aggregation reactions were aliquoted after certain time points from the commencement of the aggregation reactions and added to a 10% (w/w) ratio to the fresh aggregation reaction of NM monomers (2.5 μM) in seeded assembly buffer (40 mM Hepes–KOH pH 7.4, 150 mM KCl, 20 mM MgCl_2_, 1 mM DTT, 10 μM ThT, 3 mM GdmCl). The seeded aggregation reactions were kept at RT under stirring at 80 rpm using magnetic beads, and the ThT fluorescence was recorded with time.

### Dot-blot assays

Monomeric NM (2.5 μM) was aggregated in the assembly buffer (40 mM Hepes–KOH pH 7.4, 150 mM KCl, 20 mM MgCl_2_, 1 mM DTT) in the presence of Hsp104 (0.025 μM), ATP (5 mM), and ATP-regeneration system (20 mM PEP and 15 μg/ml PK) at RT under stirring at 80 rpm, and after 7 h and 30 h from the commencement of the reaction, the aliquots (2 μl) were spotted on the nitrocellulose membrane. NM monomers (2.5 μM) were also aggregated in the absence of Hsp104 and ATP for 7 h under the same conditions in the same assembly buffer and spotted (2 μl) on the nitrocellulose membrane. The blots were blocked using 3% bovine serum albumin in PBS with Tween-20 (PBST) (0.05 % Tween-20) for 1 h at RT and then probed with the primary antibody (A11;1:500) and (anti-His; 1:10,000) overnight at 4 °C. The blots were washed six times with PBST and incubated with an appropriate HRP-conjugated secondary antibody for 1 h at RT. Again, the blots were washed thrice using PBST and subsequently developed using an ECL kit.

### Estimation of the Sup35NM monomers recruited in the amyloids

Monomeric NM (2.5 μ M) were aggregated in the assembly buffer (40 mM Hepes–KOH pH 7.4, 150 mM KCl, 20 mM MgCl_2_, 1 mM DTT) without or with Hsp104 (0.025 μ M), ATP (5 mM), and the ATP-regeneration system (20 mM PEP and 15 μg/ml PK) for 6 h or 30 h, respectively, at RT under stirring at 80 rpm, and the amyloids generated in the reactions were pelleted down at 16,400 rpm for 30 min. The pellets were resuspended in 8 M urea (20 mM Tris–HCl, pH 7.4) overnight to monomerize the amyloids, and SDS-PAGE was performed. The Coomassie-stained monomeric NM band intensities were relatively estimated using the ImageJ software (www.imagej.nih.gov) concerning the band corresponding to the monomers of a known NM concentration in 8 M urea (20 mM Tris–HCl, pH 7.4) (62). To validate the occurrence of secondary nucleation, aliquots were taken from the Hsp104-mediated NM aggregation reaction after 6 h and 30 h, respectively, from the commencement of the aggregation reactions. The amyloids so formed were retrieved and then monomerized in 8 M urea (20 mM Tris–HCl, pH 7.4) following the protocol mentioned previously, and after the SDS-PAGE, the fraction of monomers recruited in the amyloids were compared in both the samples by comparing the Coomassie band intensities of the NM monomers using the ImageJ software. NM monomers (2.5 μM) were aggregated without or with 0.050 μM Hsp104 at RT and stirred at 80 rpm for 6 h or 30 h, respectively. Then, the generated NM or NM-Hsp104 fibrils were precipitated by centrifugation at 16,400 rpm for 30 min. The retrieved pellets were resuspended in 8 M urea (20 mM sodium phosphate, pH 7.4) and kept overnight before the SDS-PAGE analysis. Full gels are provided in Fig. S8.

### Thermal melting of fibrils

Monomeric NM (2.5 μM) was aggregated for 6 h or 30 h in assembly buffer (40 mM Hepes–KOH pH 7.4, 150 mM KCl, 20 mM MgCl_2_, 1 mM DTT) in the absence or presence of (0.025 μM) Hsp104 with ATP (5 mM) and the ATP-regeneration system (20 mM PEP and 15 μg/ml PK), respectively, at RT under stirring at 80 rpm using a magnetic bead for the fibril formation. Then, fibrils were passed through a 50 kDa filter to concentrate ∼20 times and eliminate the unrecruited monomers. The concentrated fibrils, with SDS-PAGE loading dye (2% SDS), were incubated at different temperatures for 5 min and then SDS-PAGE was performed. Coomassie-stained bands were quantified using ImageJ software. The band intensities were plotted against the incubation temperatures and fitted to the sigmoidal function. NM (2.5 μM) was also aggregated in the same assembly buffer, additionally having GdmCl (3 mM) in the presence of the same amount of Hsp104, ATP, and ATP-regeneration system for 6 h for the fibrillization, and the thermal melting experiment was performed on these fibrils as well. Full gels are provided in Fig. S8.

### The proteinase K digestion of fibrils

Monomeric NM (2.5 μM) was aggregated in the assembly buffer (40 mM Hepes–KOH pH 7.4, 150 mM KCl, 20 mM MgCl_2_, 1 mM DTT) in the absence or presence of Hsp104 (0.025 μM), ATP (5 mM), and ATP-regeneration system (20 mM PEP and 15 μg/ml PK) for 6 h or 30 h, respectively, to generate the fibrils, and after that, the fibrils were concentrated and freed from unrecruited monomers using a 50 kDa filter. The concentrated fibrils (in the supernatant) from both the aggregation reactions were incubated with proteinase K in multiple ratios at 37 °C for 30 min, and digestion reactions were terminated by adding SDS-PAGE loading dye and then SDS-PAGE was performed. The undigested NM monomers were also probed with (anti-His; 1:10,000) antibodies in Western blot analysis. PK (15 μg/ml) was added after fibrillation in the case of NM-only aggregation to make the reaction mixture comparable to the NM-Hsp104 aggregation reaction. Full gels are provided in Fig. S8.

### Disaggregation of fibrils

Monomeric NM (2.5 μ M) were aggregated in the assembly buffer (40 mM Hepes–KOH pH 7.4, 150 mM KCl, 20 mM MgCl_2_, 1 mM DTT, 10 μ M ThT) without or with Hsp104 (0.025 μ M), ATP (5 mM), and the ATP-regeneration system (20 mM PEP and 15 μg/ml PK) for 6 h or 30 h, respectively, at RT under stirring at 80 rpm to generate the fibrils, and after that, Hsp104 was added in both the reactions to a final concentration of 0.5 μM along with ATP (5 mM) and the ATP-regeneration system (20 mM PEP, and 15 μg/ml PK); all the components were mixed thoroughly and kept at RT under stirring at 80 rpm in the dark to avoid photobleaching. ATP was added last to avoid the disaggregation by ATP itself due to its hydrotropic property (63). A drop in the ThT fluorescence at 480 nm was recorded as a function of time that signified fibril disaggregation. Alternatively, the fibrils were also disaggregated by the ultrasonic sound (Qsonica probe sonicator) of amplitude 5 for 30 s for several pulses, and the ThT fluorescence was recorded after each 30 s pulse. Also, the fibrils were kept at RT for 24 h in the dark, static condition to record the decrease in the ThT fluorescence due to the autodisaggregation. The percentage of disaggregation was estimated using ([Initial ThT fluorescence intensity - final ThT fluorescence intensity]/Initial ThT fluorescence intensity) × 100%. Normalization was performed with respect to the initial ThT fluorescence intensity for the disaggregation kinetics.

### Steady-state fluorescence measurements

Steady-state fluorescence measurements for Trp mutants of Sup35NM were performed in NM and NM-Hsp104 amyloid states using the FluoroMax-4 spectrofluorometer (Horiba Jobin Yvon). For recording the fluorescence spectra, the mutants were excited at 295 nm, where the excitation and emission slit widths were 1.75 and 6 nm, respectively. Concomitantly, steady-state anisotropy measurements were performed by setting the excitation wavelength at 295 nm and emission wavelength at 330 nm with an integration time of 2 s and a bandpass of 2.5 nm and 10 nm, respectively. All of the aforementioned measurements were done at 24 ± 1 °C, and steady-state fluorescence measurements were estimated by using the parallel and perpendicular intensities taking into consideration the G-factor as shown below (37).

(1) r = (I - I⊥G)/(I + 2I⊥G)

### AFM

Monomeric NM (2.5 μ M) were aggregated in the assembly buffer (40 mM Hepes–KOH pH 7.4, 150 mM KCl, 20 mM MgCl_2_, 1 mM DTT) without or with Hsp104 (0.025 μ M), ATP (5 mM), and the ATP-regeneration system (20 mM PEP and 15 μg/ml PK), and samples were aliquoted after different time points from the commencement of the reactions for imaging. For some samples for AFM, Hsp104 and ATP were introduced after 1 h from the commencement of reaction, and samples were aliquoted after indicated time points after. For AFM imaging, the mica was freshly cleaved and washed with filtered water. Twenty microliter of the sample was deposited on mica. The sample was incubated for 5 min. The mica was washed with 100 μl of filtered water twice, followed by drying under a gentle nitrogen stream. The AFM images were acquired on Innova atomic force microscope (Bruker) using the NanoDrive (v8.03) software (www.bruker.com) (64). The images were processed and analyzed using the WSxM 5.0 Develop software (www.wsxm.eu). The height profiles were plotted using Origin 9.65 (www.originlab.com). The length of the amyloids was accessed using the Mountains software courtesy of Digital Surf.

### Statistical analysis

All the experiments were repeated at least three times, and the data are represented as mean ± SD showing the scattered data points from independent experimental replicates. The statistical significance analysis was performed by carrying out one-way ANOVA tests, and the *p*-values were reported in the figure legends. All the data analysis, data fitting (adjusted R^2^ > 0.95), and data plotting was performed using Origin 9.6.

## Data availability

All data described are contained within this paper.

## Supporting information

This article contains supporting information.

## Conflict of interest

The authors declare that they have no conflicts of interest with the contents of this article.

## References

[1] Chiti F., Dobson C.M. (2017). Protein misfolding, amyloid formation, and human disease: a summary of progress over the last decade. Annu. Rev. Biochem..

[2] Ke P.C., Zhou R., Serpell L.C., Riek R., Knowles T.P.J., Lashuel H.A. (2020). Half a century of amyloids: past, present and future. Chem. Soc. Rev..

[3] Scheckel C., Aguzzi A. (2018). Prions, prionoids and protein misfolding disorders. Nat. Rev. Genet..

[4] Brundin P., Melki R. (2017). Prying into the prion hypothesis for Parkinson’s disease. J. Neurosci..

[5] Goedert M., Masuda-Suzukake M., Falcon B. (2017). Like prions: the propagation of aggregated tau and α-synuclein in neurodegeneration. Brain.

[6] Walker L.C., Schelle J., Jucker M. (2016). The prion-like properties of amyloid- b assemblies: implications for Alzheimer ’ s disease. Cold Spring Harb. Perspect. Biol..

[7] Masnata M., Sciacca G., Maxan A., Bousset L., Denis H.L., Lauruol F. (2019). Demonstration of prion-like properties of mutant huntingtin fibrils in both *in vitro* and *in vivo* paradigms. Acta Neuropathol..

[8] Costa D.C.F., de Oliveira G.A.P., Cino E.A., Soares I.N., Rangel L.P., Silva J.L. (2016). Aggregation and prion-like properties of misfolded tumor suppressors: is cancer a prion disease?. Cold Spring Harb. Perspect. Biol..

[9] Hou Y., Dan X., Babbar M., Wei Y., Hasselbalch S.G., Croteau D.L. (2019). Ageing as a risk factor for neurodegenerative disease. Nat. Rev. Neurol..

[10] Wolff S., Weissman J.S., Dillin A. (2014). Differential scales of protein quality control. Cell.

[11] Hartl F.U., Bracher A., Hayer-Hartl M. (2011). Molecular chaperones in protein folding and proteostasis. Nature.

[12] Hipp M.S. (2019). The proteostasis network and its decline in ageing. Nat. Rev. Mol. Cell Biol..

[13] Glover J.R., Lindquist S. (1998). Hsp104, Hsp70, and Hsp40: a novel chaperone system that rescues previously aggregated proteins. Cell.

[14] Sweeny E.A., Tariq A., Gurpinar E., Go M.S., Sochor M.A., Kan Z.Y. (2020). Structural and mechanistic insights into Hsp104 function revealed by synchrotron X-ray footprinting. J. Biol. Chem..

[15] Yuan A.H., Garrity S.J., Nako E., Hochschild A. (2014). Prion propagation can occur in a prokaryote and requires the ClpB chaperone. Elife.

[16] Mogk A., Bukau B., Kampinga H.H. (2018). Cellular handling of protein aggregates by disaggregation machines. Mol. Cell.

[17] Howard M.K., Sohn B.S., von Borcke J., Xu A., Jackrel M.E. (2020). Functional analysis of proposed substrate-binding residues of Hsp104. PLoS One.

[18] Tittelmeier J., Sandhof C.A., Ries H.M., Druffel-Augustin S., Mogk A., Bukau B. (2020). The HSP110/HSP70 disaggregation system generates spreading-competent toxic α-synuclein species. EMBO J..

[19] Feleciano D.R., Juenemann K., Iburg M., Brás I.C., Holmberg C.I., Kirstein J. (2019). Crosstalk between chaperone-mediated protein disaggregation and proteolytic pathways in aging and disease. Front. Aging Neurosci..

[20] Chernova T.A., Chernoff Y.O., Wilkinson K.D. (2019). Yeast models for amyloids and prions: environmental modulation and drug discovery. Molecules.

[21] Ter-Avanesyan M.D., Dagkesamanskaya A.R., Kushnirov V.V., Smirnov V.N. (1994). The SUP35 omnipotent suppressor gene is involved in the maintenance of the non-Mendelian determinant [psi+] in the yeast Saccharomyces cerevisiae. Genetics.

[22] Glover J.R., Kowal A.S., Schirmer E.C., Patino M.M., Liu J.J., Lindquist S. (1997). Self-seeded fibers formed by Sup35, the protein determinant of [PSI+], a heritable prion-like factor of S. cerevisiae. Cell.

[23] Grimminger-Marquardt V., Lashuel H.A. (2010). Structure and function of the molecular chaperone Hsp104 from yeast. Biopolymers.

[24] Desantis M.E., Shorter J. (2012). Hsp104 drives “protein-only” positive selection of sup35 prion strains encoding strong [PSI+]. Chem. Biol..

[25] Klaips C.L., Hochstrasser M.L., Langlois C.R., Serio T.R. (2014). Spatial quality control bypasses cell-based limitations on proteostasis to promote prion curing. Elife.

[26] Zhao X., Rodriguez R., Silberman R.E., Ahearn J.M., Saidha S., Cummins K.C. (2017). Heat shock protein 104 (Hsp104)-mediated curing of [PSI+] yeast prions depends on both [PSI+] conformation and the properties of the Hsp104 homologs. J. Biol. Chem..

[27] Moriyama H., Edskes H.K., Wickner R.B. (2000). [URE3] prion propagation in Saccharomyces cerevisiae : requirement for chaperone Hsp104 and curing by overexpressed chaperone Ydj1p. Mol. Cell. Biol..

[28] Greene L.E., Zhao X., Eisenberg E. (2018). Curing of [PSI+] by Hsp104 overexpression: clues to solving the puzzle. Prion.

[29] Ness F., Ferreira P., Cox B.S., Tuite M.F. (2002). Guanidine hydrochloride inhibits the generation of prion “seeds” but not prion protein aggregation in yeast. Mol. Cell. Biol..

[30] Park Y.N., Morales D., Rubinson E.H., Masison D., Eisenberg E., Greene L.E. (2012). Differences in the curing of [PSI+] prion by various methods of Hsp104 inactivation. PLoS One.

[31] Taguchi H., Kawai-Noma S. (2010). Amyloid oligomers: diffuse oligomer-based transmission of yeast prions. FEBS J..

[32] Xue W.F., Hellewell A.L., Hewitt E.W., Radford S.E. (2010). Fibril fragmentation in amyloid assembly and cytotoxicity: when size matters. Prion.

[33] Marchante R., Beal D.M., Koloteva-Levine N., Purton T.J., Tuite M.F., Xue W.F. (2017). The physical dimensions of amyloid aggregates control their infective potential as prion particles. Elife.

[34] Breydo L., Uversky V.N. (2015). Structural, morphological, and functional diversity of amyloid oligomers. FEBS Lett..

[35] Shorter J., Lindquist S. (2004). Hsp104 catalyzes formation and elimination of self-replicating Sup35 prion conformers. Science.

[36] Adamcik J., Mezzenga R. (2018). Amyloid polymorphism in the protein folding and aggregation energy landscape. Angew. Chem. - Int. Ed..

[37] Lakowicz J.R. (2006).

[38] Kayed R., Head E., Sarsoza F., Saing T., Cotman C.W., Necula M. (2007). Fibril specific, conformation dependent antibodies recognize a generic epitope common to amyloid fibrils and fibrillar oligomers that is absent in prefibrillar oligomers. Mol. Neurodegener..

[39] Krishnan R., Goodman J.L., Mukhopadhyay S., Pacheco C.D., Lemke E.A., Deniz A.A. (2012). Conserved features of intermediates in amyloid assembly determine their benign or toxic states. Proc. Natl. Acad. Sci. U. S. A..

[40] Madhu P., Mukhopadhyay S. (2020). Preferential recruitment of conformationally distinct amyloid-β oligomers by the intrinsically disordered region of the human prion protein. ACS Chem. Neurosci..

[41] Grimminger V., Richter K., Imhof A., Buchner J., Walter S. (2004). The prion curing agent guanidinium chloride specifically inhibits ATP hydrolysis by Hsp104. J. Biol. Chem..

[42] Shorter J., Lindquist S. (2006). Destruction or potentiation of different prions catalyzed by similar Hsp104 remodeling activities. Mol. Cell.

[43] Sweeny E.A., Jackrel M.E., Go M.S., Sochor M.A., Razzo B.M., DeSantis M.E. (2015). The Hsp104 N-terminal domain enables disaggregase plasticity and potentiation. Mol. Cell..

[44] Linse S. (2017). Monomer-dependent secondary nucleation in amyloid formation. Biophys. Rev..

[45] Tanaka M., Chien P., Naber N., Cooke R., Weissman J.S. (2004). Conformational variations in an infectious protein determine prion strain differences. Nature.

[46] Kushnirov V.V., Dergalev A.A., Alexandrov A.I. (2020). Proteinase K resistant cores of prions and amyloids. Prion.

[47] Rufo C.M., Moroz Y.S., Moroz O.V., Stöhr J., Smith T.A., Hu X. (2014). Short peptides self-assemble to produce catalytic amyloids. Nat. Chem..

[48] Narang D., Swasthi H.M., Mahapatra S., Mukhopadhyay S. (2017). Site-speci Fi C fluorescence depolarization kinetics distinguishes the amyloid folds responsible for distinct yeast prion strains. J. Phys. Chem. B.

[49] Jain N., Mukhopadhyay S. (2015).

[50] Majumdar A., Mukhopadhyay S. (2018). Fluorescence depolarization kinetics to study the conformational preference, structural plasticity, binding, and assembly of intrinsically disordered proteins. Met. Enzymol..

[51] Jucker M., Walker L.C. (2018). Propagation and spread of pathogenic protein assemblies in neurodegenerative diseases. Nat. Neurosci..

[52] Krzewska J., Melki R. (2006). Molecular chaperones and the assembly of the prion Sup35p, an *in vitro* study. EMBO J..

[53] Arimon M., Grimminger V., Sanz F., Lashuel H.A. (2008). Hsp104 targets multiple intermediates on the amyloid pathway and suppresses the seeding capacity of A β fibrils and protofibrils. J. Mol. Biol..

[54] Jain N., Narang D., Bhasne K., Dalal V., Arya S., Bhattacharya M. (2016). Direct observation of the intrinsic backbone torsional mobility of disordered proteins. Biophys. J..

[55] Krishnan R., Lindquist S.L. (2005). Structural insights into a yeast prion illuminate nucleation and strain diversity. Nature.

[56] Helsen C.W., Glover J.R. (2012). A new perspective on Hsp104-mediated propagation and curing of the yeast prion [PSI+]. Prion.

[57] Stein K.C., True H.L. (2014). Extensive diversity of prion strains is defined by differential chaperone interactions and distinct amyloidogenic regions. PLoS Genet..

[58] Huang V.J., Stein K.C., True H.L. (2013). Spontaneous variants of the [RNQ+] prion in yeast demonstrate the extensive conformational diversity possible with prion proteins. PLoS One.

[59] Cobb N.J., Apostol M.I., Chen S., Smirnovas V., Surewicz W.K. (2014). Conformational stability of mammalian prion protein amyloid fibrils is dictated by a packing polymorphism within the core region. J. Biol. Chem..

[60] Ayers J.I., Schutt C.R., Shikiya R.A., Aguzzi A., Kincaid A.E., Bartz J.C. (2011). The strain-encoded relationship between PrPSc replication, stability and processing in neurons is predictive of the incubation period of disease. PLoS Pathog..

[61] Sweeny E.A., Desantis M.E., Shorter J. (2011). Purification of Hsp104, a protein disaggregase. J. Vis. Exp..

[62] Schneider C.A., Rasband W.S., Eliceiri K.W. (2012). HISTORICAL commentary NIH image to ImageJ : 25 years of image analysis. Nat. Met..

[63] Patel A., Malinovska L., Saha S., Wang J., Alberti S., Krishnan Y. (2017). Biochemistry: ATP as a biological hydrotrope. Science.

[64] Horcas I., Fernández R., Gómez-Rodríguez J.M., Colchero J., Gómez-Herrero J., Baro A.M. (2007). Wsxm: a software for scanning probe microscopy and a tool for nanotechnology. Rev. Sci. Instrum..

