# Supplementary material for: Substoichiometric Hsp104 regulates the genesis and persistence of self-replicable amyloid seeds of Sup35 prion domain
Source: J Biol Chem. 2022 Jun 14;298(8):102143. doi: 10.1016/j.jbc.2022.102143 (PMC9304785; doi:10.1016/j.jbc.2022.102143)
Supplement: Supporting information [file mmc1.pdf]

Supporting Information

Sub-stoichiometric Hsp104 regulates the genesis and persistence of self-replicable amyloid seeds of Sup35 prion domain

Sayanta Mahapatra,[1,2] Anusha Sarbahi,[1,2] Priyanka Madhu,[1,3,5] Hema M. Swasthi,[1,3,6] Abhishek Sharma,[4] Priyanka Singh,[4] and Samrat Mukhopadhyay[1,2,3,*]

[1]Centre for Protein Science, Design and Engineering, [2]Department of Biological Sciences, and [3]Department of Chemical Sciences, Indian Institute of Science Education and Research (IISER) Mohali, Punjab, India. [4]CSIR-Institute of Microbial Technology (IMTECH), Chandigarh, India

[5]Present address: Department of Chemistry, Uppsala University, Sweden
[6]Present address: Molecular, Cellular, and Developmental Biology, University of Michigan, USA

*To whom correspondence should be addressed. Email: mukhopadhyay@iisermohali.ac.in

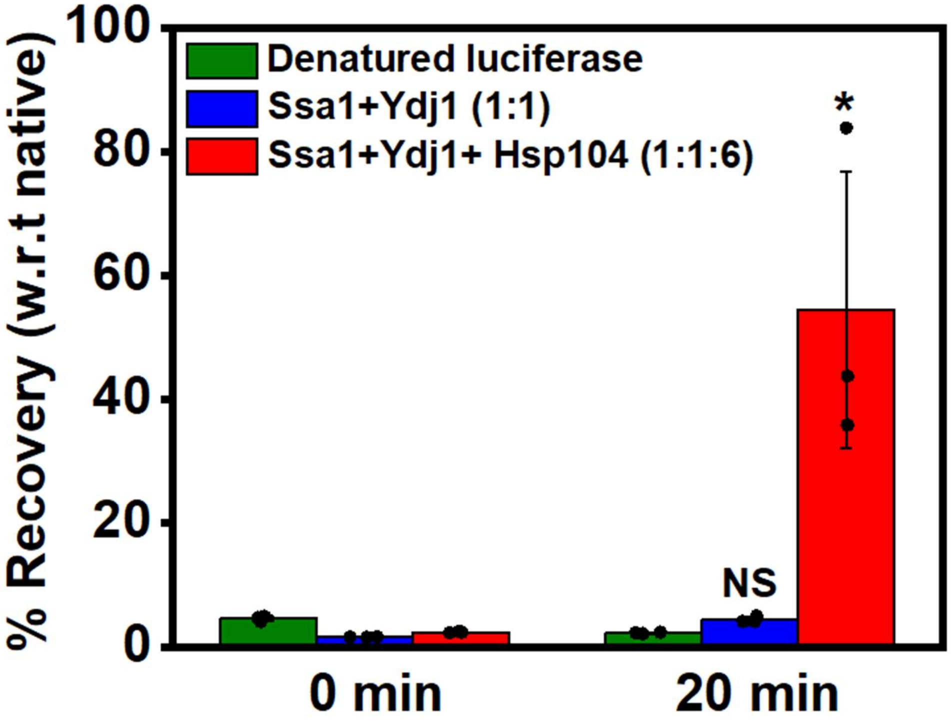

Figure S1. Luciferase reactivation assay using denatured Luciferase (80 nM) in the absence or presence the of Ssa1 (1 µM) and Ydj1 (1 µM), also in the presence of Ssa1 (1 µM) , Ydj1 (1 µM) and Hsp104 (6 µM) with ATP (1 mM) and ATP regeneration system. The extent of recovery of luciferase was calculated by the percentage of chemiluminescence with respect to native luciferase. Standard deviations were estimated using three independent replicates (n = 3). NS for Ssa1-Ydj1, *$P < 0.05$ for Ssa1-Ydj1-Hsp104 with respect to denatured luciferase. (One-way ANOVA)

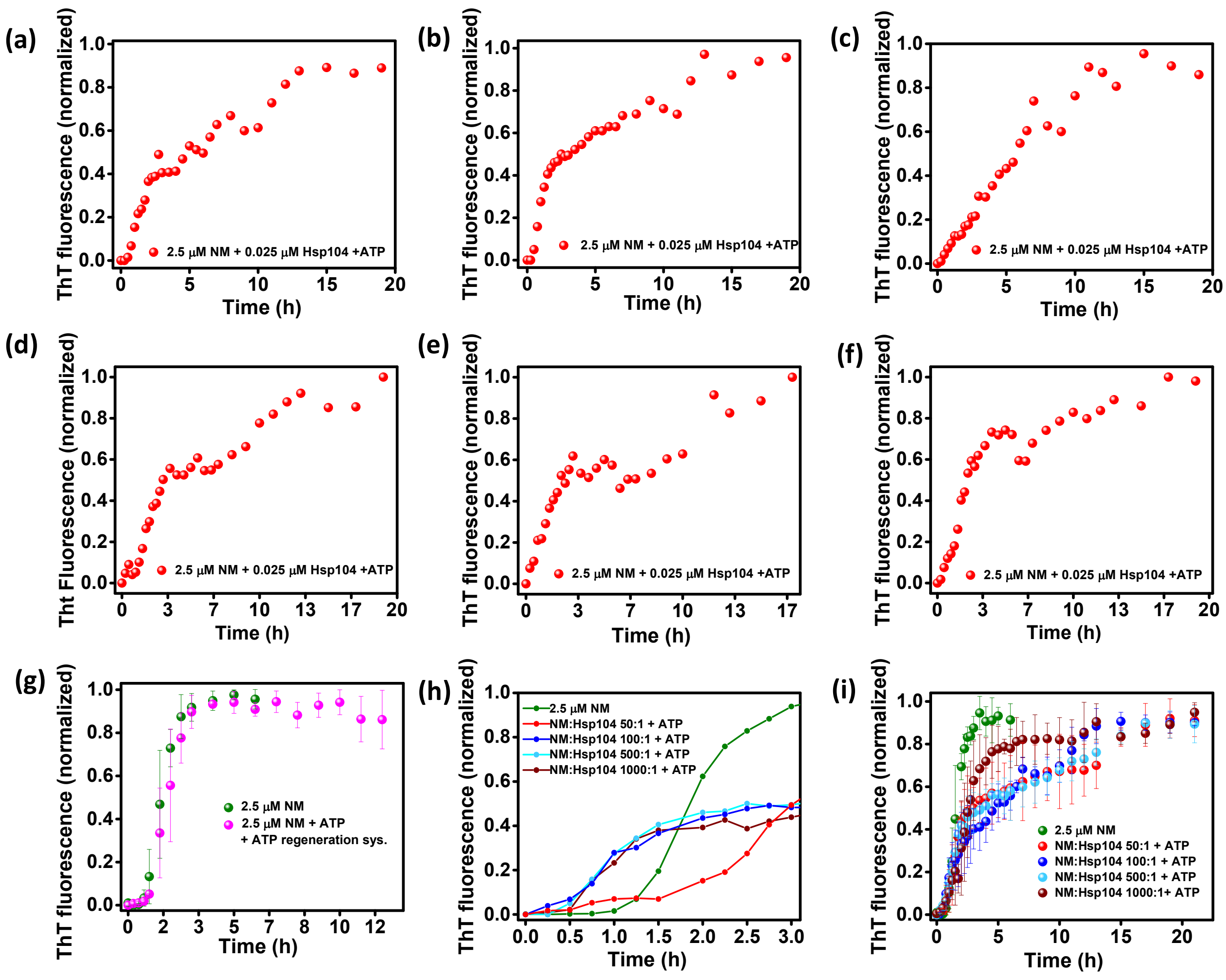

Figure S2. (a-f) Representative normalized rotated (80 rpm) thioflavin-T (ThT) fluorescence kinetics of NM (2.5 µM) with Hsp104 (0.025 µM), plus ATP (5 mM) during amyloid formation at room temperature. (g) Normalized ThT fluorescence kinetics of rotated (80 rpm) NM (2.5 µM) aggregation without or with ATP (5 mM) and ATP regeneration system [Phosphoenolpyruvate (20 mM) and pyruvate kinase (15 µg/ml)]. (h,i) Normalized ThT fluorescence kinetics of NM (2.5 µM) without or with Hsp104 and ATP (5 mM) during amyloid formation at room temperature and 80 rpm showing the (h) first 3 h (representative kinetics) and (i) 20 h of aggregation. Standard deviations were estimated from three independent experiments (n = 3).

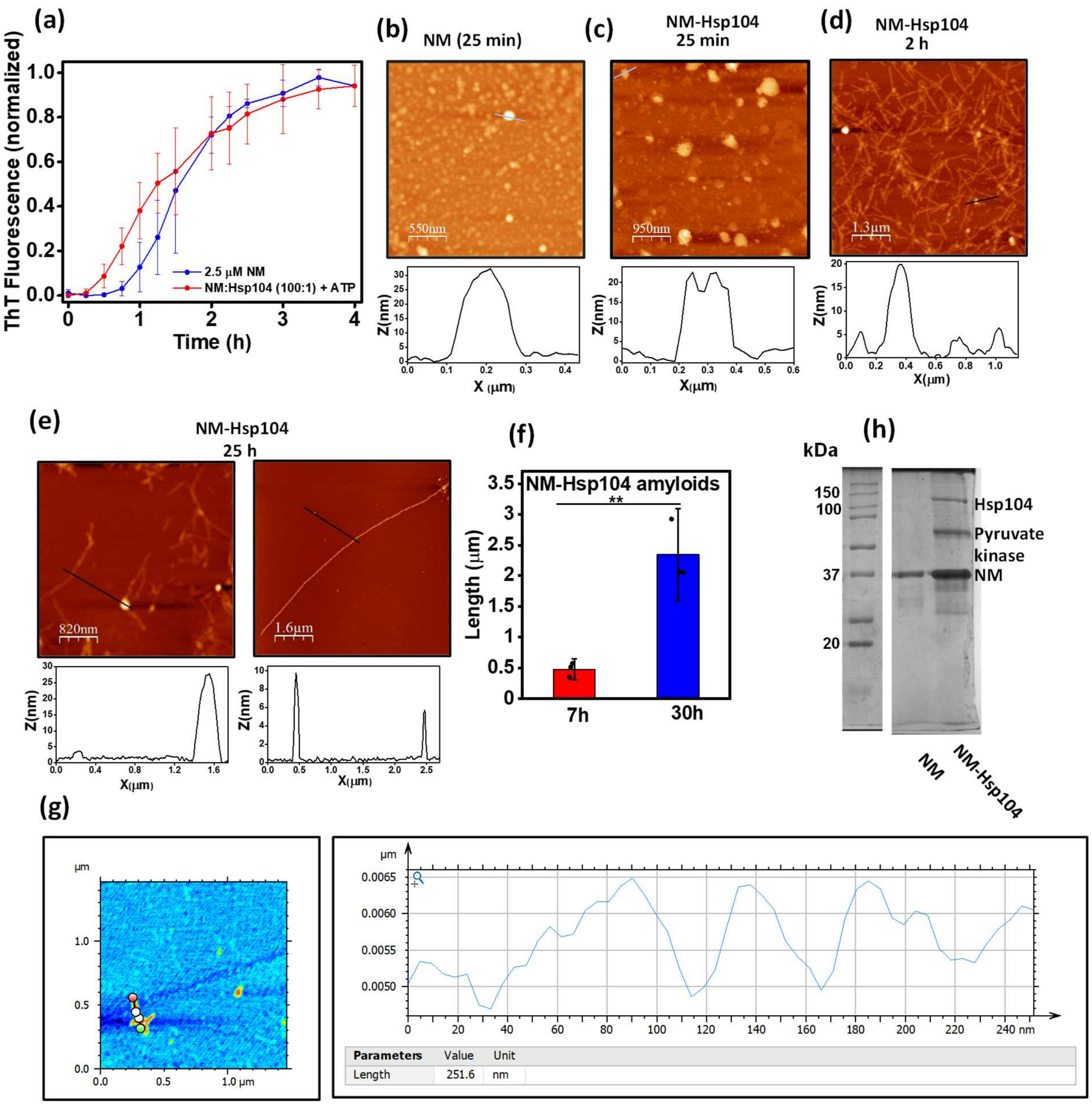

Figure S3. (a) Normalized ThT fluorescence kinetics of rotated (80 rpm) NM (2.5 μM) aggregation of first 4 h without or with Hsp104 (0.025 μM), plus ATP (5 mM) during amyloid formation at room temperature. Standard deviations were estimated from three independent experiments (n = 3). (b,c) AFM images of the (b) NM oligomers (c) NM-Hsp104 oligomers with the height ~ 25 nm in the absence or presence of Hsp104 (0.025 μM) and ATP, respectively, after 25 min. (d,e) AFM images of the (d) protofibrils with the height ~ 7 nm after 2 h, and (e) oligomers, protofibrils, and fibrils formed after 25 h of aggregation with the height ~ 25 nm, ~ 5 nm, and ~ 10 nm, respectively, in the presence of Hsp104 (0.025 μM) and ATP. (f) The length estimation of NM amyloids (2.5 μM monomers) formed in the presence of Hsp104 (0.025 μM), plus ATP (5 mM) from AFM images by using MountainsSPIP 8 software from three independent experimental replicates (n = 3), $^{**}P < 0.01$ (One-way ANOVA). (g) A representative length analysis plot. (h) The fibrils formed from monomeric NM (2.5 μM) without or with Hsp104 (0.050 μM), plus ATP after 6 h or 30 h, respectively, were retrieved by centrifugation at 16,400 rpm for 30 min. The pellets were resuspended in 8 M Urea (20 mM Tris, pH7.4) and incubated overnight prior to SDS-PAGE analysis. The uncropped gel is provided in Figure S8h.

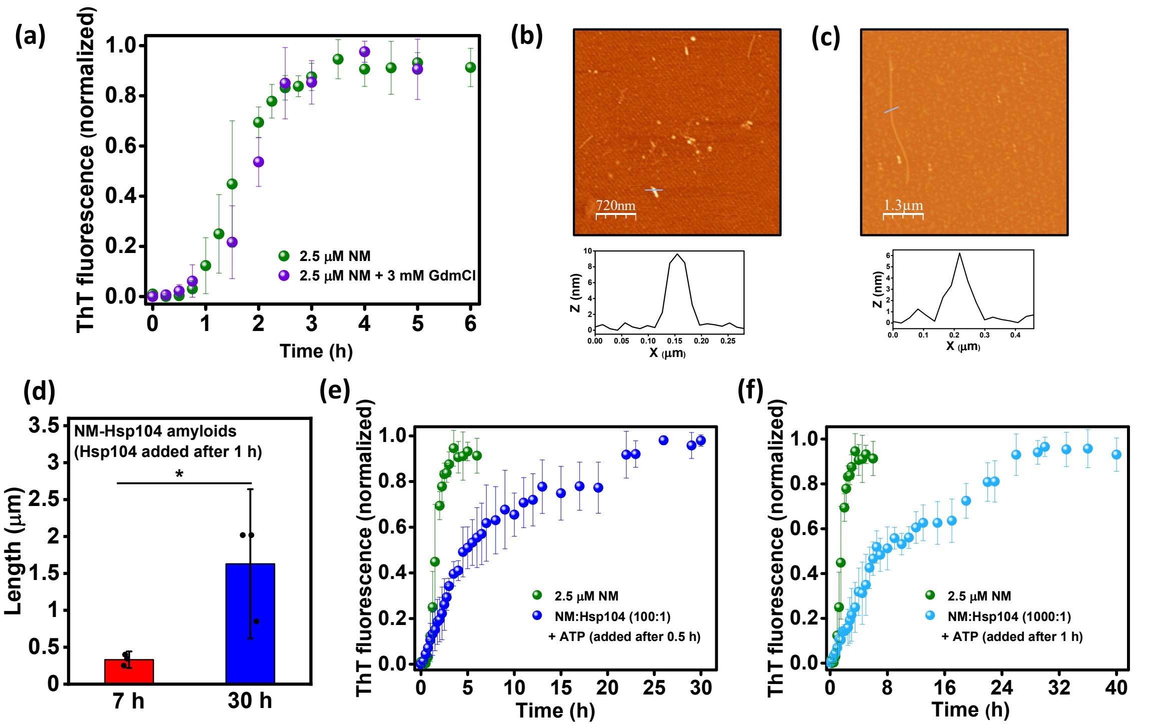

Figure S4. (a) Normalized ThT fluorescence kinetics of rotated (80 rpm) NM (2.5 µM) aggregation without or with GdmCl (3 mM) during amyloid formation. All the standard deviations were calculated from three independent experiments (n = 3). (b,c) AFM images of NM amyloids (2.5 µM monomers) showing (b) the protofibrils after 7 h with the height of ~ 8 nm and (c) the fibrils after 30 h with the height of ~ 6 nm, in the presence of Hsp104 (0.025 µM), plus ATP both were introduced after 1 h from the commencement of the reactions. (d) The length of the NM-Hsp104 (Hsp104 and ATP were introduced after 1 h) protofibrils and fibrils were estimated from AFM images of three independent replicates (n = 3) using MountainsSPIP 8; *$P < 0.05$ (One-way ANOVA). (e) Normalized ThT fluorescence kinetics of rotated (80 rpm) NM (2.5 µM) aggregation without or with Hsp104 (0.025 µM), plus ATP, introduced after 0.5 h from the commencement of the reaction. Standard deviations were calculated from three independent experiments (n = 3). (f) Normalized ThT fluorescence kinetics of rotated (80 rpm) NM (2.5 µM) aggregation without or with Hsp104 (0.0025 µM), plus ATP, introduced after 1 h from the commencement of the reaction. Standard deviations were calculated from three independent experiments (n = 3).

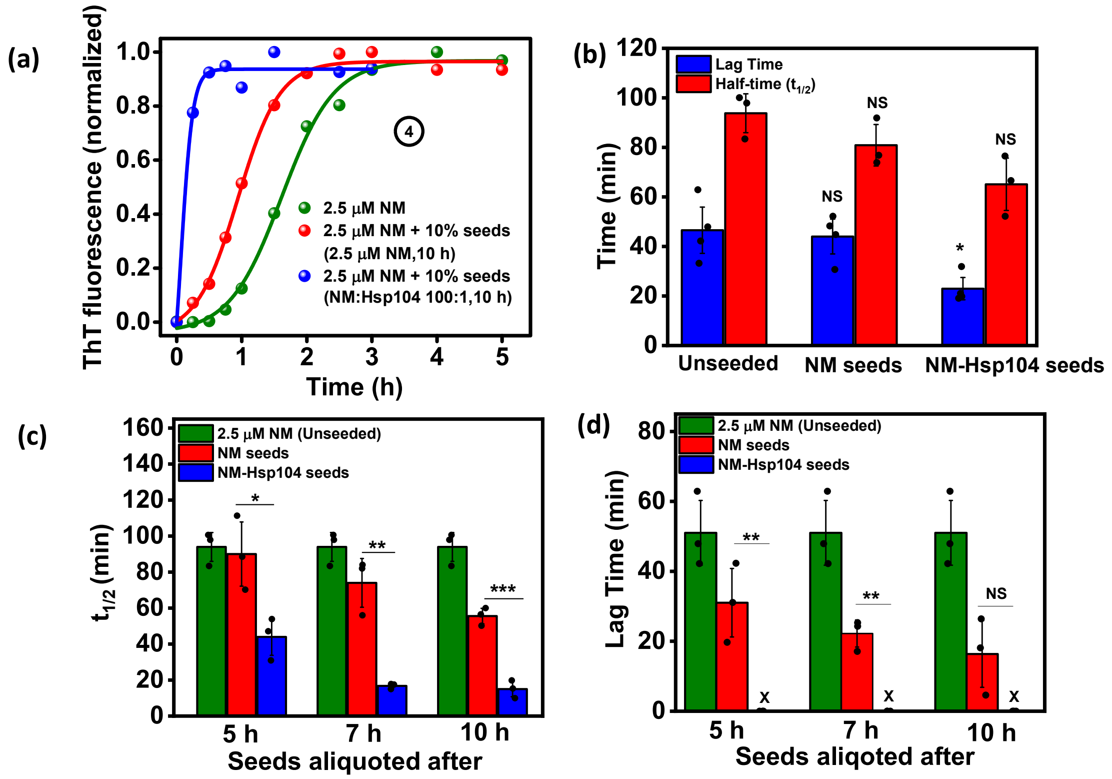

Figure S5. (a) Representative normalized ThT fluorescence kinetics of rotated (80 rpm) NM (2.5 μM) aggregation without or with 10% (w/w) seeds of NM-Hsp104 or NM aggregation that were aliquoted after 10 h from the commencement of the aggregation reaction. (b) From Figure 3b, lag times and $t_{1/2}$ of the unseeded aggregation reactions and aggregation reactions seeded with amyloids from NM or NM-Hsp104 aggregation reaction were recovered from the fitted sigmoidal plots. Standard deviations were estimated from four independent experiments (n = 4). NS, **P* < 0.05 for the lag times of NM seeds and NM-Hsp104 seeds, respectively, compared to the unseeded NM aggregation. Standard deviations were estimated from three independent experiments (n = 3). NS, NS for the $t_{1/2}$ of NM seeds and NM-Hsp104 seeds, respectively, compared to the unseeded NM aggregation. (c) From Figure 3c,d, and S5a, $t_{1/2}$ of the unseeded or aggregation reactions seeded with amyloids from NM or NM-Hsp104 aggregation reactions formed after 5 h, 7 h, and 10 h from the commencement of reactions. The standard deviations were estimated from three independent experiments (n = 3) and **P* < 0.05, ***P* < 0.01, ****P* < 0.001, respectively for 5 h, 7 h, and 10 h NM-Hsp104 seeds compared to NM-only seeds (One-way ANOVA). (d) From Figure 3c,d, and S5a, lag time of the unseeded or aggregation reactions seeded with amyloids from NM or NM-Hsp104 aggregation reactions formed after 5 h, 7 h, and 10 h from the commencement of reactions. The standard deviations were estimated from three independent experiments (n = 3) and ***P* < 0.01, ***P* < 0.01, NS, respectively, for 5 h, 7 h, and 10 h NM-Hsp104 seeds compared to NM seeds (One-way ANOVA).

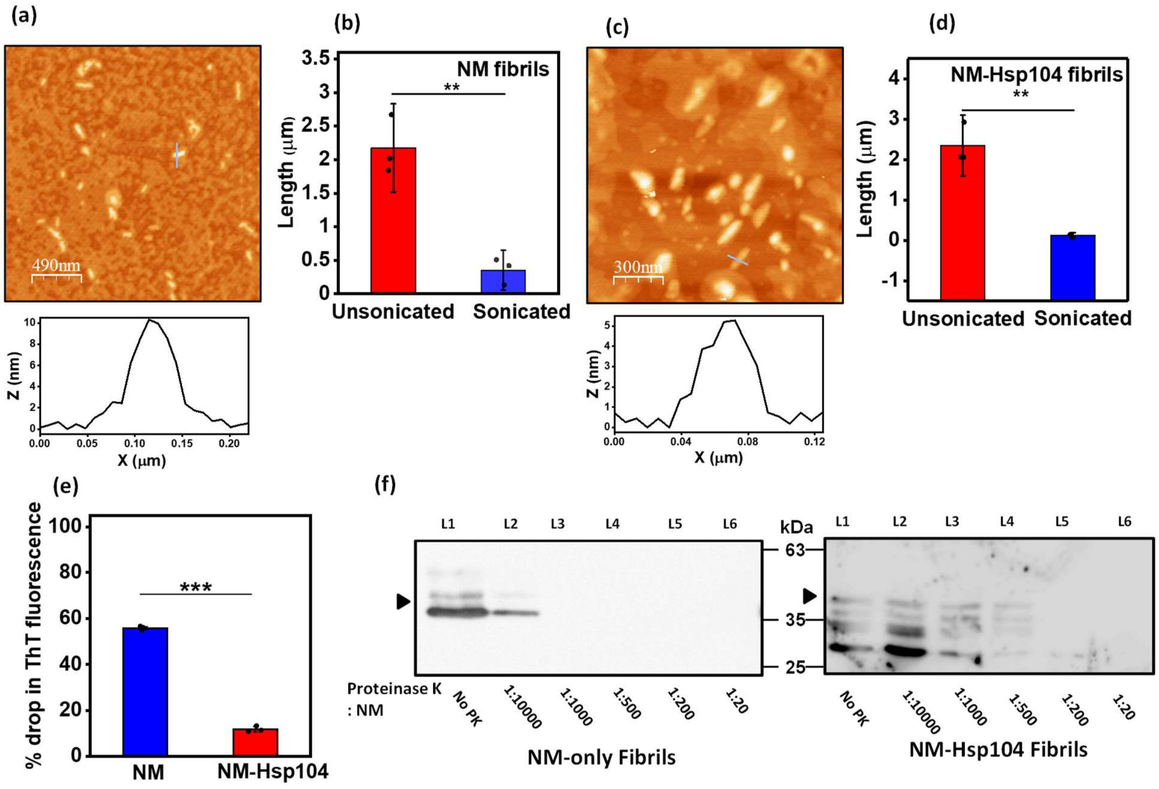

Figure S6. (a) AFM images of sonicated (4 x 30 sec-pulse of amplitude 5) NM fibrils showing the decrease in the length of amyloids in Figure S6b from three different independent experimental replicates (n = 3) compared to the unsonicated, intact fibrils, $^{**}P < 0.01$ (One-way ANOVA). (c) AFM images of sonicated (4 x 30 sec-pulse of amplitude 5) NM-Hsp104 fibrils showing the decrease in the length of amyloids in Figure S6d from three different independent experimental replicates (n = 3) compared to the unsonicated, intact fibrils, $^{**}P < 0.01$ (One-way ANOVA). (e) Auto-disaggregation of NM and NM-Hsp104 fibrils by keeping the fibrils at room temperature for 24 h in unagitated conditions and the drop in the ThT fluorescence was estimated. Standard deviations were calculated from three independent experiments (n = 3), $^{***}P < 0.001$ (One-way ANOVA). (f) The concentrated fibrils formed from monomeric NM (2.5 µM) without or with Hsp104 (0.025 µM), plus ATP then incubated at 37 °C for 30 min with multiple concentrations of proteinase K followed by the Western blot analysis with anti-His antibody. The bands corresponding to the NM monomers are marked with black triangles.

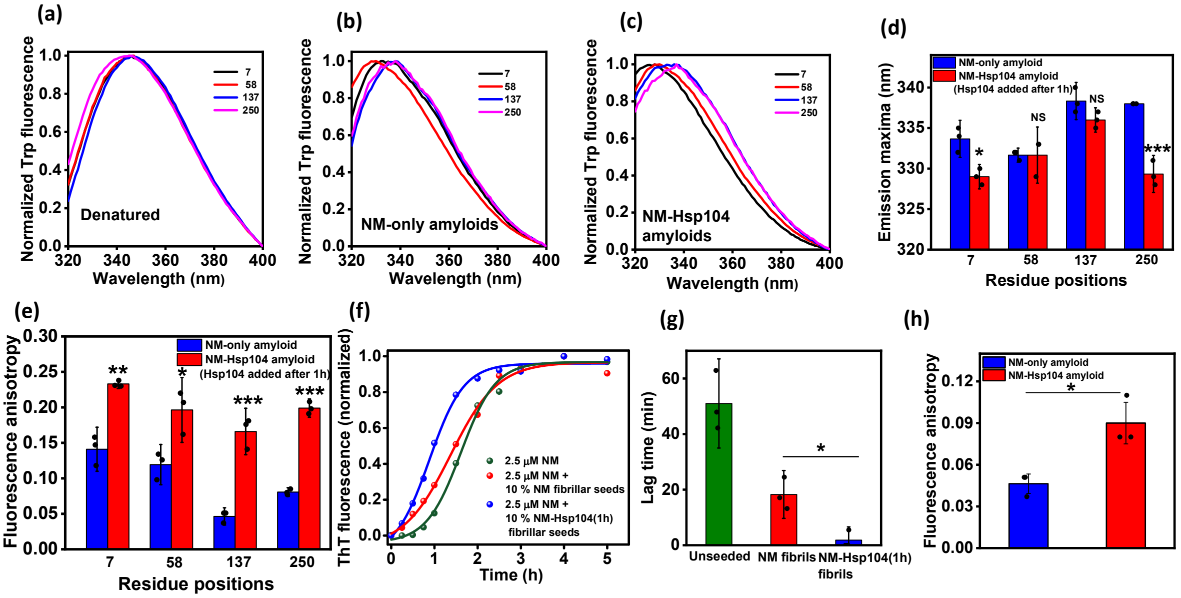

Figure S7. (a-c) Normalized Trp fluorescence spectra of different residues in (a) denatured (b) NM-only amyloid (c) NM-Hsp104 amyloid states (NM:Hsp104 100:1). (d) Trp emission maxima of different residue positions in two different amyloid forms, NM and NM-Hsp104 (NM:Hsp104 100:1, Hsp104 and ATP were introduced after 1 h). The excitation and emission slit widths were 1.75 and 6 nm, respectively. Standard deviations were estimated from three different experimental replicates (n = 3), **P* < 0.05, NS, NS, ****P* < 0.001 (One-way ANOVA) for locations 7,58,137,250, respectively, with respect to the NM-only amyloids. (e) Steady-state fluorescence anisotropies of different residue reactions (n = 3), **P* < 0.05 compared to the NM fibrils. positions in two amyloid states NM and NM-Hsp104 (NM:Hsp104 100:1, Hsp104 and ATP were introduced after 1 h). Standard deviations were estimated from three different experimental replicates (n = 3), ***P* < 0.01, **P* < 0.05, ****P* < 0.001, ****P* < 0.001 (One-way ANOVA) for locations 7,58,137,250, respectively, with respect to the NM-only amyloids. (f) Representative normalized ThT fluorescence kinetics of rotated (80 rpm) NM (2.5 μM) aggregation without seeds or with 10% (w/w) NM fibrillar seeds and NM-Hsp104 (Hsp104 and ATP were introduced after 1 h) fibrillar seeds in assembly buffer containing GdmCl (3 mM). (g) The lag times of all the aggregation reactions were retrieved. Standard deviations were estimated from three independent unseeded or seeded aggregation (h) Steady-state fluorescence anisotropy of at residue position 137 in two amyloid states (NM and NM-Hsp104, NM: Hsp104 1000:1). Standard deviation was estimated from three independent replicates (n = 3), **P* < 0.05 (One-way ANOVA) for location 137, with respect to the NM-only amyloids.

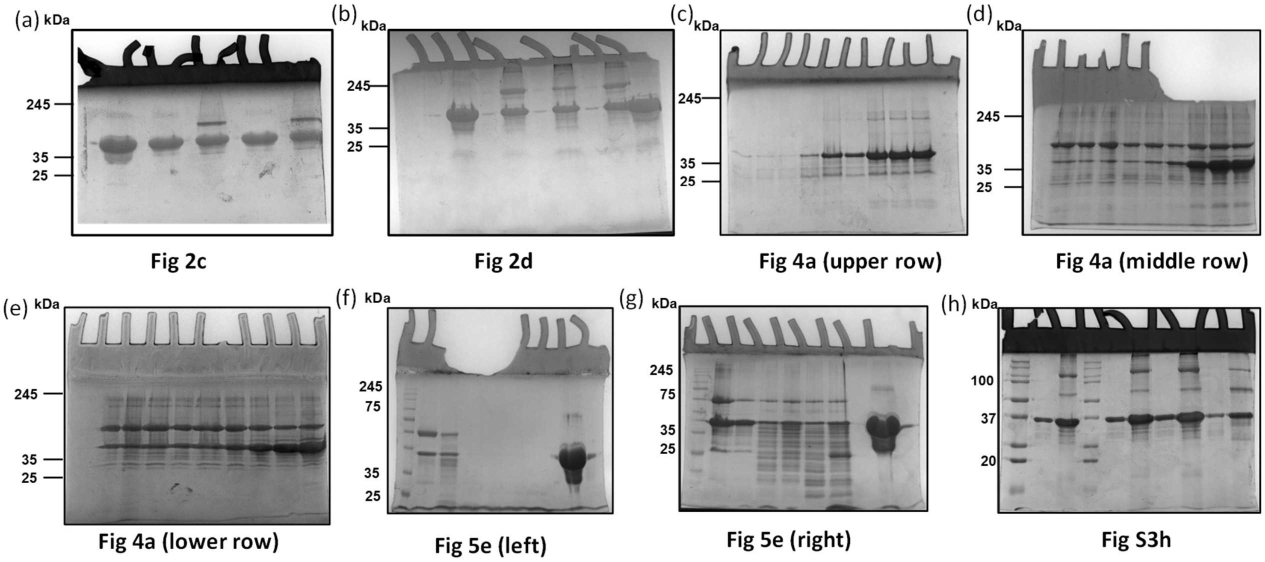

Figure S8. Full uncropped gels used in respective figures as indicated below each gel image.
